# Correlation inference attacks against machine learning models

**DOI:** 10.1126/sciadv.adj9260

**Published:** 2024-07-10

**Authors:** Ana-Maria Creţu, Florent Guépin, Yves-Alexandre de Montjoye

**Affiliations:** ^1^ EPFL, Lausanne, Switzerland.; ^2^ Imperial College London, London, UK.

## Abstract

Despite machine learning models being widely used today, the relationship between a model and its training dataset is not well understood. We explore correlation inference attacks, whether and when a model leaks information about the correlations between the input variables of its training dataset. We first propose a model-less attack, where an adversary exploits the spherical parameterization of correlation matrices alone to make an informed guess. Second, we propose a model-based attack, where an adversary exploits black-box model access to infer the correlations using minimal and realistic assumptions. Third, we evaluate our attacks against logistic regression and multilayer perceptron models on three tabular datasets and show the models to leak correlations. We lastly show how extracted correlations can be used as building blocks for attribute inference attacks and enable weaker adversaries. Our results raise fundamental questions on what a model does and should remember from its training set.

## Teaser

Correlation leakage in machine learning models and its use as a building block in attribute inference attacks are studied.

## INTRODUCTION

Machine learning (ML) is widely used today by companies, governments, and researchers to learn from data how to automate processes and make decisions. ML models are, for example, used for medical diagnosis (*1*), language translation (*2*), speech recognition and synthesis (*3*), chatbots, content moderation (*4*), drug discovery (*5*), content retrieval (*6*), and personalized services.

While models are often described as solutions to general tasks, e.g., detecting early-stage cancer in young patients, learning is performed on specific finite datasets (*7*). The understanding of the relationship between a model and the specific dataset that it was trained on is both a fundamental question, from a learning theory perspective, and a very important one in practice, for instance, to evaluate the circumstances in which a model can be used. The extreme cases would be, on the one hand, an ideal hypothetical case where the model remembers nothing more from the dataset what is necessary for its intended goal: inferring an output variable based on the input—what we write M(*x*) ≃ *P*(*Y* ∣ *X* = *x*). The other extreme would be a case where the model encodes the entire dataset and, when asked for a prediction, outputs the label of the closest record in its training set. Both are obviously extreme hypothetical cases and real models are likely to fall somewhere in between. For instance, models have until recently been smaller in size than their training dataset, suggesting that some level of learning happens. On the other hand, million- and even billion-parameter models have today become standard, e.g., in large language models (*8*), suggesting that models might remember quite a lot about their training dataset, potentially bringing us slightly closer to the encoded case.

The intuitive way to study the relationship between a model and its training dataset could be to track how the model “came to be,” i.e., how the final parameters of the model are computed, step by step, from the training dataset. This is, however, increasingly difficult. Learning from a dataset today involves performing a complex, iterative, and stochastic procedure, over random subsets of the dataset (so-called mini-batches) to update, sometimes, billions of parameters using gradient descent. In practice, the resulting model is closer to a “black box”: a set of parameters whose precise relationship to the training dataset is largely untraceable.

Trained models have instead been studied in a post hoc fashion, trying to understand what they can reveal about their training dataset, and thus what they “remember” (*9*). More specifically, inference attacks, in the form of games, have been used to study whether specific pieces of information can be extracted from a trained model, showing that the model “remembers” them (*10*–*15*).

So far, inference attacks have, however, mostly focused on inferring the presence or absence of a given record in the dataset, a membership inference attack (MIA) (*15*, *16*). In an MIA, the adversary aims to infer, better than random, whether a specific record was part of a dataset used to train the model. A large body of work has used MIAs to build a robust understanding of the conditions under which information about individual records is more likely to leak, e.g., when a model is overfitted or trained on a small dataset (*17*–*24*).

Membership is, however, only one of the characteristics of the training dataset that can be remembered by a model. We here exclude the hypothetical case where membership attacks would achieve perfect or close to perfect accuracy and records can be enumerated. This would lead to the dataset being reconstructable and any other properties to be directly computed. Current MIAs are, however, very far from reaching a perfect accuracy across a large range of records. We do not believe that near-future attacks are likely to reach close to perfect accuracy in general either.

In particular, there has been little work studying whether models encode dataset-level characteristics. An important line of research, for instance, aims to audit ML models to study biases or to better understand the range of values within which a model can operate, a crucial question in practice (*25*). From a privacy perspective, dataset-level characteristics could reveal sensitive information (*10*, *26*, *27*). For instance, a scoring model for depression could remember that patients living in the inner city are more likely to have used illegal substances. The answer to the question of what a model does or even should remember from its training dataset is not simple. One could argue that the ideal “fully generalizable” model should learn nothing more than the intended probability of the output label given an input sample M(*x*) ≃ *P*(*Y* ∣ *X* = *x*). Models today and in the near future, however, result from minimizing a loss function computed over a specific finite dataset of samples from the underlying dataset distribution *P*(*X*, *Y*) (*7*). The dataset distribution *P*(*X*, *Y*) and, through marginalization, *P*(*X*), may very well end up influencing the final model in practice.

Contribution: In this work, we study a previously unknown type of leakage in ML models: the leakage of correlations between input variables of a tabular training dataset by proposing what is, to our knowledge, the first correlation inference attack (CIA) against ML models. We argue that (i) the leakage of correlations by an ML model can be seen as a privacy violation, which goes beyond the model’s intended purpose and can lead to individual harm (see Discussion for examples); (ii) correlations can be used as building blocks for other attacks on individual privacy such as attribute inference attacks (AIAs), particularly enabling weaker adversaries (see Attribute inference attack methodology); and (iii) CIAs are different from previously studied property inference attacks (PIAs), because PIAs target something else and rely on strong assumptions that make them inapplicable to CIAs (see Related work for a detailed comparison).

We propose two different CIAs. First, a model-less attack that uses knowledge of the correlations between the input variables *X* and the output variable *Y*, which we call correlation constraints, to predict the correlation between two input variables ρ(*X*_1_, *X*_2_). We argue that the correlation constraints are typically known to or inferrable by the attacker. Our attack exploits the spherical parameterization of correlation matrices, imposing bounds on the correlation coefficients, to make an informed guess. This first attack also acts as a strong baseline, allowing us to correctly quantify the leakage caused by the ML model, and not by the adversary’s prior knowledge about the dataset. We then propose a second model-based attack that exploits access to an ML model trained on the dataset of interest. Our model-based attack uses a meta-classifier relying only on synthetic data, markedly reducing the strength of the adversary compared to that in the literature. To generate synthetic data, we combine Gaussian copula–based generative modeling as our prior with carefully adapted procedures for sampling correlation matrices under constraints.

We then study the performance of our model-based attack on synthetic data under three different attack scenarios. By order of increasing adversary knowledge, we consider an adversary that knows (i) the correlation constraints relating only to the target variables, (ii) all the input variables, or (iii) the correlations between all the variables except for the target correlation, ρ(*X*_1_, *X*_2_). Across scenarios, we first show our model-based attack to consistently strongly outperform the model-less attack, showing that ML models such as logistic regressions (LRs) and multilayer perceptrons (MLPs) leak information about the correlations between their input variables *X*. Second, we show how mitigations such as limiting the number of queries that can be made to the model or their precision fail to prevent the attack. Third, we show that, expectedly, ensuring that the model is differentially private (DP) does not prevent our attack and extend our attack to and validate it on three real-world datasets. We lastly argue that dataset-level statistics can be building blocks for other attacks, particularly enabling weaker adversaries. To illustrate our argument, we propose an AIA exploiting the correlations extracted from the model and show it to significantly outperform previous attacks on real-world datasets. We make our source code allowing to reproduce the results of our experiments available at https://github.com/computationalprivacy/ml-correlation-inference.

## RESULTS

### Correlation inference threat model

We consider an ML model M_T_ trained on a target dataset *D*_T_ consisting of *m* records *z*^1^, …, *z^m^*. Each *z^i^* is a sample of *n* − 1 input variables *X*_1_, …, *X*_*n*−1_ and one output variable *Y*. We assume variables to be continuous and real-valued and denote by *F_i_*(*x*) = Pr (*X_i_* ≤ *x*) the one-way marginal of the *i*th input variable and by *F_n_*(*y*) the one-way marginal of the output variable. For simplicity, we consider M_T_ to be a binary classifier whose goal is to infer if *Y* > *c*, where *c* is a task-specific threshold such as the average or the median value. Our attack can, however, be easily generalized to multi-class classification and regression tasks.

Our adversary aims to infer the Pearson correlation coefficient ρ(*X*_1_, *X*_2_) between two input variables *X*_1_ and *X*_2_ given access to the ML model M_T_. Also called linear correlation, the Pearson correlation coefficient measures the strength of the linear association between two variables and is widely used by researchers and data scientists across many domains (*28*, *29*). We call ρ(*X*_1_, *X*_2_) the target correlation and *X*_1_ and *X*_2_ the target variables.

We make two assumptions on the adversary knowledge on the data distribution:

1) Knowledge of one-way marginals. First, we assume the adversary to have knowledge of the one-way marginals of all the variables *F_i_*(*x*), *i* = 1, …, *n* − 1 and *F_n_*(*y*). These are typically released, e.g., in research papers or to comply with legal requirements such as the European Union AI Act (*30*), and their knowledge is a common assumption in the literature (*13*, *14*, *27*).

2) Knowledge of some correlations. Second, we assume the adversary to have access to the linear correlations between some of the variables. More specifically, we define the partial knowledge of the adversary as a set *P* of pairs such that, for every (*i*, *j*) ∈ *P*, the adversary has the knowledge of the ground-truth values ρ(*X_i_*, *X_j_*) (with *X_n_* = *Y*). As ρ(*X_i_*, *X_j_*) = ρ(*X_j_*, *X_i_*), by symmetry, we assume that *P* consists of ordered pairs, i.e., if (*i*, *j*) ∈ *P*, then necessarily *i* < *j*.

We consider three different attack scenarios:

(S1) The adversary knows the correlations between the target variables and the output variable, namely, ρ(*X*_1_, *Y*) and ρ(*X*_2_, *Y*), i.e., *P* = {(1, *n*), (2, *n*)}, but not ρ(*X_i_*, *X_j_*) for 1 ≤ *i* < *j* ≤ *n* − 1 nor ρ(*X_i_*, *Y*) for *i* ≥ 3.

(S2) The adversary knows the correlations between all the input variables and the output variable: [ρ(*X_i_*, *Y*)]_*i* = 1, …, *n*−1_, i.e., *P* = {(*i*, *n*) : *i* = 1, …, *n* − 1}, but the adversary does not know ρ(*X_i_*, *X_j_*) for 1 ≤ *i* < *j* ≤ *n* − 1.

(S3) The adversary knows all the correlations between the variables, except for the target correlation ρ(*X*_1_, *X*_2_). More specifically, the adversary knows ρ(*X_i_*, *X_j_*), ∀ 1 ≤ *i* < *j* ≤ *n* such that (*i*, *j*) ≠ (1,2), i.e., *P* = {(*i*, *j*) : 1 ≤ *i* < *j* ≤ *n*}\{(1,2)}.

Note that the three scenarios are identical for *n* = 3 variables.

We will henceforth call the known coefficients the correlation constraints.

S2 is the default scenario considered here. This choice is motivated by the fact that the relationship between the input and output variables, encoded through ρ(*X_i_*, *Y*), is unlikely to be considered a secret, because the model’s goal is to infer *Y* given *X*. We furthermore show in Discussion how these correlation constraints can be extracted from the model.

S1 is a weak adversary, motivated by our goal to measure the leakage from the model, rather than the leakage derived from constraints imposed on *X*_1_ and *X*_2_ via their association with the output variable *Y*. Our analysis shows that the correlation coefficients ρ(*X*_1_, *Y*) and ρ(*X*_2_, *Y*) restrict the range of values that the target coefficient ρ(*X*_1_, *X*_2_) can take, without the adversary even accessing the model.

S3 is the strongest, “worst-case” scenario, corresponding to an adversary having complete knowledge of the correlations, except for the target. We mostly study this scenario to evaluate how much information a very strong adversary would be able to infer.

We consider two different assumptions on the adversary access to the target ML model M_T_.

1) Model-less attack. Under this attack scenario, the adversary does not have access to the target model M_T_. This scenario mostly serves as a stronger than random baseline allowing to measure the adversary’s uncertainty on the target correlation under the prior knowledge. It is necessary to consider this scenario to properly evaluate the leakage from the model. A method for this scenario might also become an attack in situations where the model is not released, yet information about the marginals and the correlations between the input variables and the target variables is made available, e.g., as part of a research study.

2) Model-based attack. Under this attack scenario, we assume that the adversary (i) knows the model architecture and training details, e.g., number of training epochs for a neural network, allowing them to train from scratch a similar model, and (ii) has black-box query access to the target model M_T_, allowing to retrieve the output probabilities for each class M_T_(*x*) on inputs *x*.

### Attack methodology

#### Overview

We frame the correlation inference task as a classification task by dividing the range of correlations [−1,1] into *N*_B_ bins of equal length. The adversary’s goal is to infer the correlation bin to which ρ(*X*_1_, *X*_2_) belongs. When *N*_B_ = 3, the bins are [−1, −1/3), [−1/3,1/3), and [1/3,1], which we refer to as negative, low, and positive correlations, respectively [“)” denotes an open interval]. We opt for a classification rather than a regression task because it is easier to analyze the attack performance by comparing its accuracy with a random guess baseline (at 1/*N*_B_). Unless otherwise specified, we use *N_B_* = 3 classification bins in our empirical evaluation, as the correlation ranges can be easily interpreted. We also discuss in the “Increasing the number of variables *n*” section and the “Real-world dataset evaluation” section the results of our attack for *N*_B_ = 5 bins.

At a high level, our methodology analyzes the behavior of the target model through the confidence scores returned on specific inputs, searching for patterns allowing to infer the correlations between input variables. More specifically, we train a meta-classifier to infer the correlation ρ(*X*_1_, *X*_2_) between two variables of interest in a dataset *D* based on features extracted from a model M trained on *D*. Training the meta-classifier requires a large number of models M derived from datasets *D* spanning a wide variety of values for the unknown correlations ρ(*X_i_*, *X_j_*), where (*i*, *j*) ∉ *P*, while matching the correlation constraints ρ(*X_i_*, *X_j_*) for every (*i*, *j*) ∈ *P*. Generating datasets satisfying this requirement on their correlations is a key technical challenge our work addresses.

#### Correlation matrices

To formalize this requirement on the linear relationships between the *n* variables in a dataset, we use the concept of correlation matrix. This is the *n* × *n* real-valued matrix *C* of Pearson correlation coefficients between all the pairs of variables: *c*_*i*,*j*_ = ρ(*X_i_*, *X_j_*), *i*, *j* = 1, …, *n*. Note that we use lowercase notation to denote matrix coefficients. Let C be the set of valid correlation matrices. C consists of all *n* × *n* real-valued matrices satisfying the following properties:

(P1) All elements are valid correlations, i.e., real values between −1 and 1: −1 ≤ *c*_*i*,*j*_ ≤ 1, ∀ 1 ≤ *i*, *j* ≤ *n*.

(P2) Diagonal entries are equal to 1, i.e., there is perfect correlation between a variable and itself: *c*_*i*,*i*_ = 1, ∀ 1 ≤ *i* ≤ *n*.

(P3) The matrix is symmetric, i.e., the correlation between *X_i_* and *X_j_* is the same as the correlation between *X_j_* and *X_i_*: *c*_*i*,*j*_ = *c*_*j*,*i*_.

(P4) The matrix is positive semi-definite: *x^T^Cx* ≥ 0, ∀ *x* ∈ ℝ*^n^*.

The spherical parameterization of correlation matrices (*31*) provides a principled and effective approach for sampling valid correlation matrices *C*. This approach crucially relies on the Cholesky decomposition of *C* = *BB^T^*, which, as we describe next, can be used to generate valid correlation matrices by sampling its correlation coefficients one by one, conditionally on the correlation constraints. More specifically, because *C* is positive semi-definite, it has a Cholesky decomposition *C* = *BB^T^*, where *B* is lower triangular with nonnegative diagonal entries: *b*_*i*,*j*_ = 0,1 ≤ *i* < *j* ≤ *n* and *b*_*i*,*i*_ ≥ 0, *i* = 1, …, *n*. Furthermore, the coefficients of matrix *B* can be expressed using spherical coordinates, as follows

$$B=\left(\begin{array}{ccccc} 1 & 0 & 0 & \cdots & 0\\ \text{cos}\theta_{21} & \text{sin}\theta_{21} & \ddots & \ddots & \vdots \\ \text{cos}\theta_{31} & \text{cos}\theta_{32}\text{sin}\theta_{31} & \text{sin}\theta_{32}\text{sin}\theta_{31} & \ddots & \vdots \\ \vdots & \vdots & \vdots & \ddots & \vdots \\ \text{cos}\theta_{n1} & \text{cos}\theta_{n2}\text{sin}\theta_{n1} & \text{cos}\theta_{n3}\text{sin}\theta_{n2}\cdot \text{sin}\theta_{n1} & \cdots & \prod_{j-1}^{n-1}‍\text{sin}\theta_{\mathrm{nj}} \end{array}\right)$$ (1)

This is because the diagonal of *C* consists of ones, i.e., 1 = *c*_*i*,*i*_ = (*BB^T^*)_*i*,*i*_ $=\sum_{j=1}^{n}‍b_{i,j}^{2}=\sum_{j=1}^{i}‍b_{i,j}^{2}$ , which means that each vector (*b*_*i*,1_, …, *b*_*i*,*i*_) ∈ ℝ*^i^* has an $L_{2}$ norm of 1 and can, therefore, be expressed using spherical coordinates, i.e., ∃θ_*i*,*j*_ ∈ [0, π),1 ≤ *i* < *j* < *n*. Note that as expected, for the symmetric matrix *C*, only $\frac{n\times \left(n-1\right)}{2}$ parameters, called angles, are sufficient to characterize the set of valid correlation matrices.

A key property exploited by both our model-less and model-based attacks is that the first column of *B* is identical to the first column of *C*. By reordering, without loss of generality, the variables as *Y*, *X*_1_, …, *X*_*n*−1_, all the correlation constraints appear on the first column as free parameters under scenarios S1 and S2. We will use this property to sample the other correlation coefficients conditionally on the correlation constraints (algorithm S2 and Algorithm 1). We will further show how sampling can be done even in scenario S3 (Algorithm 2), where the correlation constraints cannot be expressed using only the free parameters of the first column. We start by describing the model-less attack, to build intuition into how this property is useful for sampling correlation matrices under constraints.


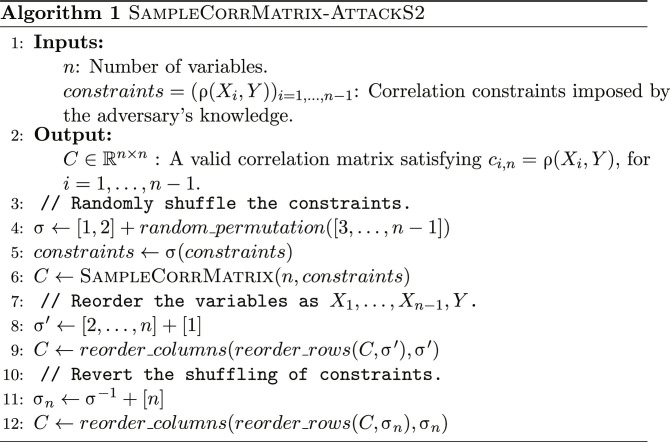


#### Model-less attack

Our model-less attack leverages the intuition that the adversary’s knowledge of some correlations allows to derive theoretical limits on the range spanned by the other values. This allows the adversary to make a better than random guess about the target correlation ρ(*X*_1_, *X*_2_) without access to the model. The model-less attack acts as a strong baseline for any attack using access to the model, allowing to correctly quantify the leakage caused by the model.

Given the correlation constraints (*c*_*i*,*j*_)_(*i*,*j*) ∈ *P*_, we call matching set *C_P_* the set of valid correlation matrices satisfying them: *C_P_* = {*C*′ ∈ C: ∀ (*k*, *l*) ∈ *P*, *c*′_*k*,*l*_ = *c*_*k*,*l*_}. Because of the correlation constraints, the range of values *R* that can be taken by *c*_1,2_ is a subinterval of [−1,1]: *R* = [inf_*C*′∈ *C_P_*_*c*′_1,2_, sup_*C*′∈ *C_P_*_*c*′_1,2_] ≔ [*m*_1_, *m*_2_]. Our model-less attack outputs the majority bin over this interval. To avoid introducing biases in the evaluation, we assume the uniform prior over this interval and return the classification bin which is covered in the largest proportion by [*m*_1_, *m*_2_] (see section S1 for details).

As an example, consider the case *n* = 3 and an adversary having knowledge of ρ(*X*_1_, *Y*) and ρ(*X*_2_, *Y*) [corresponding to *P* = {(1,2), (1,3)}]. We assume, for simplicity, that the correlation matrix is computed over *Y*, *X*_1_, *X*_2_ in this order. Developing the computation *C* = *BB^T^* yields the following correlation coefficients

$$\begin{array}{c} c_{2,1}=\text{cos}\;\theta_{2,1}=\rho \left(X_{1},Y\right)\\ c_{2,1}=\text{cos}\;\theta_{3,1}=\rho \left(X_{2},Y\right)\\ c_{3,2}=\text{cos}\;\theta_{2,1}\text{cos}\;\theta_{3,1}+\text{sin}\;\theta_{2,1}\text{sin}\;\theta_{3,1}\text{cos}\;\theta_{3,2}=\rho \left(X_{1},Y_{2}\right) \end{array}$$ (2)

Because θ_2,1_ and θ_3,1_ are fixed, the only degree of freedom left on the unknown ρ(*X*_1_, *X*_2_) is θ_3,2_. Any choice of θ_3,2_ ∈ [0, π) yields a valid correlation matrix. The unknown ρ(*X*_1_, *X*_2_) can thus take any value between cos θ_2,1_ cos θ_3,1_ − sin θ_2,1_ sin θ_3,1_ = cos (θ_2,1_ + θ_3,1_) and cos θ_2,1_ cos θ_3,1_ + sin θ_2,1_ sin θ_3,1_ = cos (θ_2,1_ − θ_3,1_) but cannot fall outside the interval determined by these endpoints. Depending on the values of the constraints, this interval might be much smaller than [−1,1], thereby reducing the uncertainty of the adversary.

**Fig. 1.**
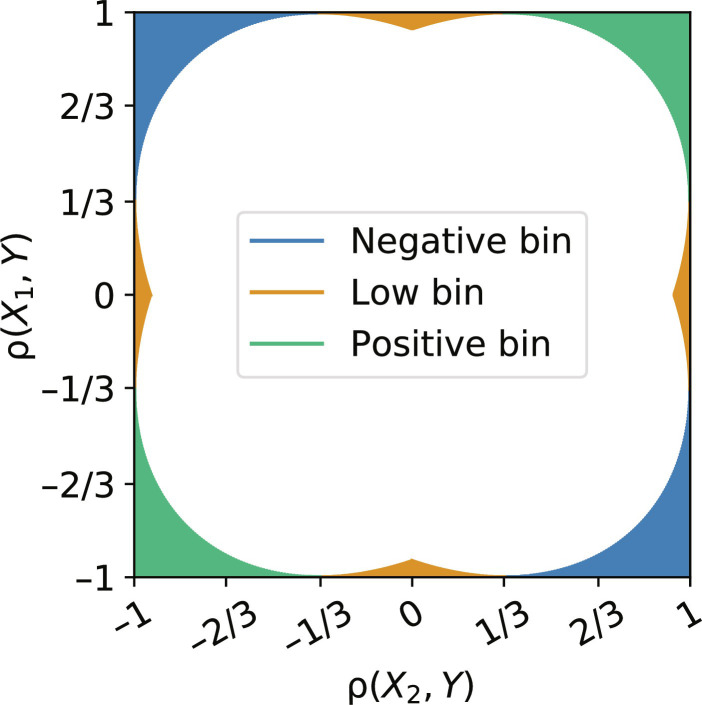
Illustration of correlation constraints [ρ(*X*_1_, *Y*), ρ(*X*_2_, *Y*)] that lead to only one possible bin for the unknown correlation ρ(*X*_1_, *X*_2_). Here, we show results for *N*_B_ = 3 possible bins.

Figure 1 shows that some constraints yield a theoretical interval *R*, which is strictly included in one of the *N*_B_ = 3 classification bins. For such constraints, the adversary can infer the target correlation bin with certainty. These constraints are of the two types. First, constraints that are close to 1 in absolute value result in high ρ(*X*_1_, *X*_2_) belonging to the positive, green bin, when they have the same sign and to the negative, blue bin, when they have opposite signs. Second, when one of the constraints is close to 0, while the other is close to 1 in absolute value, ρ(*X*_1_, *X*_2_) is necessarily in the low, orange bin. We refer the reader to section S2 for a precise, mathematical description of these regions.

This interval can be computed analytically or empirically, by sampling a sufficient number of correlation matrices satisfying the correlation constraints. We here use the latter approach, as our model-based attack also requires generating valid correlation matrices satisfying the constraints given by the adversary knowledge. We refer the reader to section S6.4 for an analysis of the impact of the number of samples on the model-less attack performance.

#### Generating correlation matrices under constraints

To generate valid correlation matrices satisfying the correlation constraints, we adapt an algorithm developed by Numpacharoen and Atsawarungruangkit (*32*). The goal of the original algorithm, which we include in section S3 (see algorithm S1), is to sample valid correlation matrices (under no constraints). The insight of the authors is that each correlation coefficients *c*_*i*,*j*_ can be expressed as *c*_*i*,*j*_ = *m*_*i*,*j*_ + cos θ_*i*,*j*_*l*_*i*,*j*_, where *m*_*i*,*j*_ and *l*_*i*,*j*_ only depend on θ_*p*,*q*_ for 1 ≤ *p* ≤ *i* and 1 ≤ *q* < *j*. Algorithm S1 uses this insight to generate valid correlation matrices by sampling the correlations in order, from top to bottom (*i* = 1, …, *n*) and from left to right (*j* = 1, …, *n*), uniformly within the bounds *m*_*i*,*j*_ ± *l*_*i*,*j*_ derived from the values previously sampled. The formulas on *m*_*i*,*j*_ and *l*_*i*,*j*_ can be derived (supposing without loss of generality that *i* ≥ *j*) by developing the computation of *c*_*i*,*j*_ = (*BB^T^*)_*i*,*j*_ = *B*_*i*,1:*j*−1_(*B*_*j*,1:*j*−1_)*^T^* + *b*_*i*,*j*_*b*_*j*,*j*_ and setting $m_{i,j}=\sum_{p=1}^{j-1}‍\left(\text{cos}\;\theta_{i,p}\text{cos}\;\theta_{j,p}\prod_{q=1}^{p-1}‍\text{sin}\;\theta_{i,q}\text{sin}\;\theta_{j,q}\right)$ and $l_{i,j}=\prod_{q=1}^{j-1}‍\text{sin}\;\theta_{i,q}\text{sin}\;\theta_{j,q}$.

We adapt this algorithm to our problem by noticing that the coefficients of the first column of *B* (cos θ_*i*,1_ for *i* = 2, …, *n* − 1) are identical to those of *C* and are free parameters. We set these coefficients equal to the correlation constraints and sample the others uniformly within their bounds. We propose two adaptations of the original algorithm for attack scenarios S1 and S2, algorithm S2 and Algorithm 1, respectively. Note that correctly sampling the correlation matrices without biasing the values requires carefully reordering the variables before and after the sampling, an intricate operation that we detail in the “Generating correlation matrices under constraints” section. We further propose an algorithm to sample the unknown value ρ(*X*_1_, *X*_2_) for attack scenario S3, when all the other correlations are known (Algorithm 2).


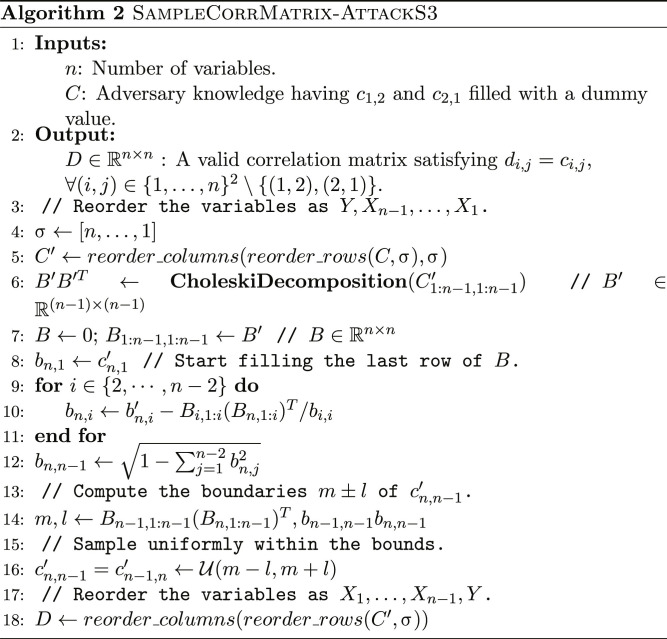


#### Model-based attack

We lastly develop a model-based attack, which trains a meta-classifier to infer the bin to which ρ(*X*_1_, *X*_2_) belongs on a labeled dataset of model and correlation pairs {*M*(*D*), ρ[*X*_1_(*D*), *X*_2_(*D*)]}. We create this labeled dataset by training models on synthetic datasets generated using the Gaussian copula generative model (*33*). We select the Gaussian copula model because it is parameterized by one-way marginals and a covariance matrix, allowing us to generate synthetic datasets that match the adversary’s knowledge about the target dataset *D*_T_ while spanning the entire range of values attainable by the unknown correlations. Using our notation, the Gaussian copula model is parameterized by (i) the marginals of the variables (*F_i_*)_1≤*i*≤*n*_ and (ii) a positive semi-definite covariance matrix Σ yielding the following joint distribution

$$\text{Pr}\left(X_{1}\leq x_{1},\ldots ,X_{n}\leq x_{n}\right)=\Phi_{\Sigma }\left\{\Phi^{-1}\left[F_{1}\left(x_{1}\right)\right],\ldots ,\Phi^{-1}\left[F_{n}\left(x_{n}\right)\right]\right\}$$ (3)

where Φ_Σ_ is the cumulative distribution function (CDF) of the Gaussian multivariate distribution 𝒩(0, Σ): $\Phi_{\Sigma }\left(x_{1},\ldots ,x_{n}\right)=\int_{-\infty }^{x_{1}}\ldots \int_{-\infty }^{x_{n}}\frac{e^{-\frac{1}{2}x^{T}\Sigma^{-1}x}}{\sqrt{{\left(2\pi \right)}^{n}\text{det}\left(\Sigma \right)}}$ and Φ is the CDF of a one-dimensional standard normal 𝒩(0,1). Algorithm S3 describes the procedure to sample from the Gaussian copulas.

Consistent with the rest of our methodology, we use correlation, rather than covariance matrices to parameterize the Gaussian copulas. Correlation matrices are a subset of covariance matrices that have only values of 1 on the diagonal.

To generate synthetic datasets *D* matching the correlation constraints and the one-way marginals of the private dataset *D_T_*, we distinguish between two cases:

1) When the variables are all standard normals, i.e., *F_i_* = Φ, *i* = 1, …, *n*, the correlations of the distribution output by the Gaussian copula are identical to the parameter Σ. We first generate a correlation matrix *C* using the algorithm corresponding to the attack scenario (e.g., Algorithm 1 for S2) and then set Σ = *C* to sample a dataset whose empirical correlation approximately match *C*.

2) When the variables have arbitrary one-way marginals, the correlations of the distribution output by the Gaussian copula parameterized by these marginals are not necessarily equal to the parameter Σ (*34*). This means that the adversary must not directly parameterize the Gaussian copula by setting Σ = *C* but, instead, must modify the correlation constraints *V* = {(*c*_T_)_*i*,*j*_, (*i*, *j*) ∈ *P*} (with *C*_T_ denoting the correlation matrix of private dataset *D*_T_) to another set of constraints *V*′, generate a correlation matrix *C*′ under the constraints given by *V*′ (instead of *V*), and only then set Σ = *C*′. *V*′ is chosen such that the empirical correlations computed on the synthetic datasets are approximately equal to *V* and thus match the correlation constraints. Algorithm 3 details a simple, yet very effective heuristic to modify the correlation constraints.

To train our meta-classifier, we first generate *K* synthetic datasets *D^k^*, *k* = 1, …, *K*. Second, we train a model M*^k^* on each dataset using the same architecture and hyperparameters as the target model, but a different seed (we refer the reader to Discussion for results of our attack when the adversary knows the seed). Last, we extract a set of features from each model in the form of confidence scores computed on a synthetic query dataset *D*_query_ generated in the same way as the other datasets, i.e., to match the adversary’s knowledge. We train the meta-classifier to infer the correlation ρ[*X*_1_(*D^k^*), *X*_2_(*D^k^*)], discretized over *B* bins, given as input the features extracted from the model M*^k^*. The complete procedure is described in the “Model-based attack” section.

### Empirical evaluation

We apply our attack to LR and MLP models, which are standard choices for learning tasks on tabular training datasets. We train the models on synthetic and real-world datasets, as described in the following sections.

Unless otherwise specified, we assume the adversary to have black-box access to a target model M_T_ trained on a target dataset *D*_T_, with the goal of inferring the correlation ρ(*X*_1_, *X*_2_). Unless otherwise specified, we consider the default attacker (S2), i.e., the attacker knows ρ(*X_i_*, *Y*)_*i*=1,…,*n*−1_.

Across experiments, we run our CIAs against the target model by, first, training a meta-classifier on features extracted from models trained on synthetic datasets satisfying the adversary’s knowledge about the target dataset *D*_T_. Second, we apply the meta-classifier to features extracted from the target model M_T_ to infer the bin to which the target correlation ρ(*X*_1_, *X*_2_) belongs. We repeat the experiment multiple times and compute the attack accuracy as the fraction of correct guesses. Complete details of the experiments are provided in section S6.

To quantify how much information is leaked by different types of models, we first compare the performances of our model-based and model-less attacks in a controlled setting. More specifically, we train the target models M_T_ on synthetic data generated using the Gaussian copula generative model. Recall that this model is parameterized by a correlation matrix, which allows us to explore the entire range that can be taken by the correlation constraints ρ(*X_i_*, *Y*)_*i* = 1,…,*n*−1_ and characterize their impact on the performance of the attack.

#### Impact of constraints for n = 3 variables

To characterize the impact of the correlation constraints on the attack accuracy, we study a simple setting in which there are only two input variables and one output variable (*n* = 3). We consider all possible ranges for the correlation constraints ρ(*X*_1_, *Y*) and ρ(*X*_2_, *Y*) by dividing the grid [−1,1] × [−1,1] into 200 × 200 equal cells. We report one accuracy for every cell [*a*_1_, *a*_2_] × [*b*_1_, *b*_2_], evaluating the performance of an attacker (model-less or model-based) at inferring the target correlation ρ(*X*_1_, *X*_2_) for datasets subject to correlation constraints belonging to that cell, i.e., such that *a*_1_ ≤ ρ(*X*_1_, *Y*) < *a*_2_ and *b*_1_ ≤ ρ(*X*_2_, *Y*) < *b*_2_.

In every cell, we draw *T*′ pairs of constraints ρ(*X*_1_, *Y*) and ρ(*X*_2_, *Y*) uniformly in [*a*_1_, *a*_2_] and [*b*_1_, *b*_2_], respectively. Each pair is used to generate, in order: (i) a target correlation matrix *C*_T_ using Algorithm 1, (ii) a target dataset *D*_T_ of 1000 samples using Gaussian copulas parameterized by *C*_T_ and standard normal one-way marginals, and (iii) a target model M_T_ trained on *D*_T_ to perform the binary classification task *Y* > 0. We make three observations regarding (i). First, because there are only *n* = 3 variables, Algorithm 1 and algorithm S2 are equivalent. Second, ρ(*X*_1_, *X*_2_) is the only correlation coefficient being generated. Third, this value is sampled uniformly over the range defined by Eq. 2 and correlation constraints ρ(*X*_1_, *Y*) and ρ(*X*_2_, *Y*). For every pair of constraints, given access to it and to M_T_, the adversary’s goal is to infer (*c*_T_)_1,2_ = ρ(*X*_1_, *X*_2_). Executing our attack independently on all the *T*′ targets in every cell would require us to generate *K* × *T*′ × 200 × 200 models to train meta-classifiers, which is computationally infeasible. Instead, in every cell, we (i) run the model-based attack by training one meta-classifier and (ii) run the model-less attack by empirically estimating, from the samples, the range of values that ρ(*X*_1_, *X*_2_) can take when the correlation constraints belong to that cell. We evaluate the attacks using fivefold cross-validation and setting *T*′ = 1500.

**Fig. 2.**
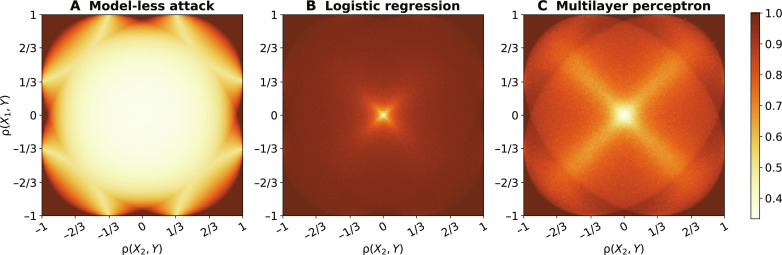
Accuracy of the CIA on *n* = 3 variables as a function of the correlation constraints ρ(*X*_1_, *Y*) and ρ(*X*_2_, *Y*). We show results of (**A**) the model-less attack and of the model-based against two different target models: (**B**) LR and (**C**) MLP. The color of each cell indicates the accuracy of our CIA applied to models trained on synthetic datasets whose correlations ρ(*X*_1_, *Y*) and ρ(*X*_2_, *Y*) belong to the region defined by the cell. There are 200 × 200 cells in total.

Figure 2A shows that an adversary with no access to the model can infer the target correlation ρ(*X*_1_, *X*_2_) 56.0% of the time, on average, over all the cells, much better than random (33.3%). These results confirm the theoretical analysis presented in Attack methodology with our model-less attack achieving perfect accuracy in the corners of the grid and the top center region (with its symmetric counterparts). They also highlight why it is essential to have a strong baseline different from the random guess baseline to quantify the leakage from the model. The accuracy of the model-less attack then slowly decreases as the constraints move to regions of higher uncertainty, in which more than one bin is possible.

Figure 2B shows that black-box access to an LR allows an adversary to greatly improve the accuracy of the attack, reaching 95.6% on average over all the cells. As expected, the model-based attack achieves perfect accuracy in the regions where the model-less attack also does. Outside these regions, the model-based attack vastly outperforms the model-less attack. Its accuracy exceeds 90% everywhere, with the exception of the cross-like region centered in the origin where the models leak less information than elsewhere. At the center of the cross [∣ρ(*X_i_*, *Y*) ∣ ≤ 0.02, *i* = 1,2], where the input variables *X*_1_ and *X*_2_ are uncorrelated with the output variable *Y*, the accuracy of our attack is 47.2%. When there is little to no correlation between the inputs and the output variable *Y* [ρ(*X_i_*, *Y*) ≃ 0], the target models do not learn much about the dataset. As for the arms of the cross, where ρ(*X*_1_, *Y*) ≃ ρ(*X*_2_, *Y*) ≪ 1, we believe that the lower attack performance is due to the theoretical interval attainable by the target correlation ρ(*X*_1_, *X*_2_) being close to [−1,1] and hence the adversary having maximum uncertainty. Even in this scenario, the leakage from the model is much higher than the baseline.

Figure 2C shows that MLP models are also vulnerable to our attack, achieving an accuracy of 82.3%, on average, over the cells. This shows that access to an MLP model greatly improves over the model-less attack but slightly less than the LR. This may seem unexpected at first, because our MLP, with its 292 learnable weights, has a much higher capacity than the LR with its 3 weights and thus a higher ability to retain information about the dataset. We believe that this gap is due to the fact that the non-convex loss used to train MLP models has a (potentially large) number of local minima (*35*). This means that the models trained by an adversary’s to generate the meta-classifier’s training dataset are unlikely to reach the same local minima as the target model, increasing the adversary’s uncertainty. We refer the reader to Discussion for results of our attack when we remove the uncertainty caused by the difference in randomness. We also find that the cross-like behavior is exacerbated on the MLP: The center of the cross is larger, reaching an average accuracy of 48.1% for ∣ρ(*X*_1_, *Y*) ∣ ≤ 0.1.

White-box results: Figs. S2 and S3 show that white-box access to the model, where the features extracted from the model include its weights, does not improve the performance of our attack. We refer the reader to section S7 in for details of the experiment and an analysis of the results.

#### Increasing the number of variables n

Next, we compare the performances of our attack against LR and MLP models when increasing the number of variables *n*, under three different attack scenarios. For each *n* = 3, …,10, we sample 1000 target correlation matrices *C*_T_ ∈ C using algorithm S1. To obtain a balanced distribution over the target correlations, we generate the value ρ(*X*_1_, *X*_2_) first, as explained in the “Generating correlation matrices under constraints” section. Then, for each *C*_T_, we sample a synthetic target dataset *D*_T_ (parameterized by *C*_T_ and standard normal marginals) and train a target model M_T_ on it.

**Fig. 3.**
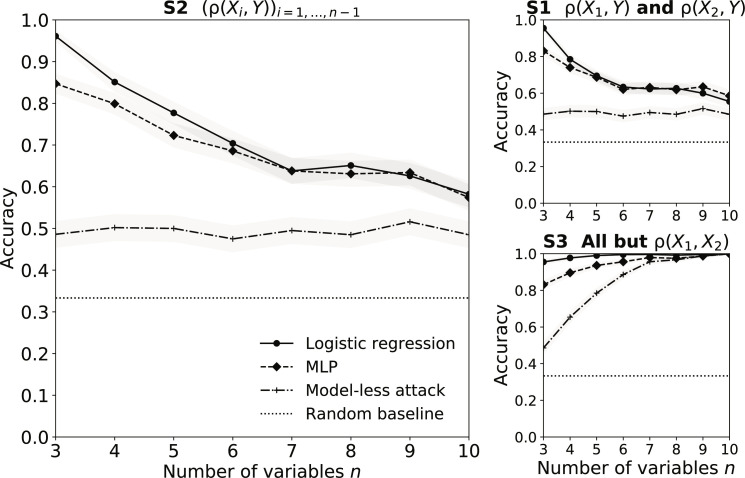
Attack accuracy for different scenarios and number of variables in the dataset *n*. We consider three different attack scenarios: (S1) the adversary knows the correlations between the target variables and the output variable ρ(*X*_1_, *Y*) and ρ(*X*_2_, *Y*), (S2) the adversary knows the correlations between all the input variables and the output variable ρ(*X_i_*, *Y*)_*i*=1,…,*n*−1_, and (S3) the adversary knows all the correlations between the variables except for the target correlation ρ(*X*_1_, *X*_2_). We compute the accuracy (with 95% confidence interval) over 1000 target models.

Scenario S2 in Fig. 3 shows that the adversary is consistently and significantly better than the model-less attack. The performance of our attack decreases slowly with the number of variables in the dataset, from 96.1% (*n* = 3) to 70.4% (*n* = 6) and 58.2% (*n* = 10) when applied to LR models, while the model-less attack has a roughly constant accuracy of 49.3% for all values of *n*. This decrease when *n* increases is expected. First, the number of unknown correlations increases quadratically with *n*, which increases the uncertainty of the adversary. Second, the more variables a model is trained on, the less likely our target variables *X*_1_ and *X*_2_ are to be useful for the prediction task at hand. This, in turn, means that the model might learn less information about ρ(*X*_1_, *X*_2_).

Figure S14 shows that, when at least one of the constraints ρ(*X*_1_, *Y*) or ρ(*X*_2_, *Y*) is large in absolute value (meaning that *X*_1_, *X*_2_, or both are good predictors of the output variable), our attack performs better. For instance, on *n* = 6 variables, our attack reaches 90.6% when max(∣ρ(*X*_1_, *Y*)∣, ∣ρ(*X*_2_, *Y*)∣) ≥ 0.8, much better than 62.7% when max(∣ρ(*X*_1_, *Y*)∣, ∣ρ(*X*_2_, *Y*)∣) ≤ 0.2. Even when both constraints are small, e.g., when max(∣ρ(*X*_1_, *Y*)∣, ∣ρ(*X*_2_, *Y*)∣) ≤ 0.2, the model-based attack can still infer ρ(*X*_1_, *X*_2_) much better than random, especially for a smaller number of variables. We discuss potential reasons for this behavior in Discussion.

The difference in vulnerability between LR and MLP models reduces as the number of variables in the dataset *n* increases, with our attack achieving similar accuracy against the two for *n* ≥ 6. We rule out the possibility of models learning the same decision boundary, as the MLP models are, on average, 1 to 4% less accurate, with the gap in accuracy increasing with *n*. We believe that this could, instead, be due to MLP models’ training algorithm becoming more stable as *n* increases, i.e., the loss having fewer local minima, as more input variables are likely to be predictive of the output *Y*.

We obtain similar trends on *N*_B_ = 5 classification bins and refer the reader to fig. S11 for detailed results. Scenario S1 in Fig. 3 shows that reducing the knowledge of correlations to only ρ(*X*_1_, *Y*) and ρ(*X*_2_, *Y*) only slightly decreases the attack accuracy. More specifically, the accuracy is 78.5% when *n* = 3 versus 85.1% for S2, 63.4% when *n* = 6 versus 70.4% for S2, and 55.5% when *n* = 10 versus 58.2% for S2. This shows that CIAs are a concern even when facing a weak adversary.

We complete our understanding of the impact of uncertainty on the attack by additionally considering a very strong adversary, who has knowledge of all the correlations but the target ρ(*X*_1_, *X*_2_). Scenario S3 in Fig. 3 shows that the accuracy of our attack now increases as *n* increases. We believe that this is because the fraction of unknown correlations now reduces quadratically with *n*. More specifically, our attack improves as *n* increases, from 95.5% (*n* = 3) to 99.5% (*n* = 6) and 99.9% (*n* = 10) on LR models. We obtain similar results on MLP models, as our attack improves from 83.1% (*n* = 3) to 95.6% (*n* = 6) and 99.9% (*n* = 10). The strongest adversary can again exploit a higher vulnerability in LR models compared to that in MLP models. The model-less attack also increases with *n* but achieves a lower accuracy than our model-based attack for *n* ≤ 8.

#### Impact of mitigations

**Fig. 4.**
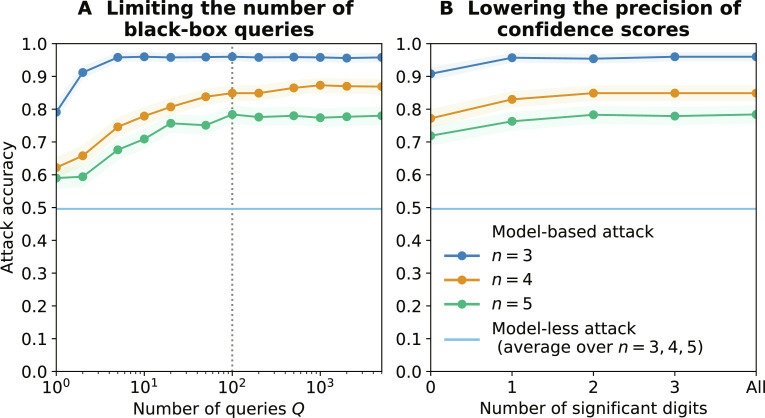
Impact of mitigations on the accuracy of our attack against LR models. We report results for two mitigations: limiting the number of black-box queries (**A**) and lowering the precision of confidence scores (**B**). We report the accuracy (with 95% confidence interval) of our model-less and model-based attacks over 1000 targets models for *n* ∈ {3,4,5} variables.

We analyze the effectiveness of three possible mitigations in preventing our CIA. Limiting the number of queries could be put in place, e.g., in the ML as a service case, by the model developer to prevent the user from extracting too much information. We vary the number of queries *Q* made to an LR from 1 to 5000 and report the attack accuracy against the same targets as before. Figure 4A shows that this measure does not prevent our attack. Only five queries are necessary to reach close to optimal accuracy for *n* = 3, 50 queries for *n* = 4, and 100 for *n* = 5. Even a single query is sufficient for the attack to reach 79.1% on *n* = 3 variables, 62.2% on *n* = 4, and 59.0% on *n* = 5, significantly higher than the model-less attack that only reaches 49.6%.

Lowering the precision of confidence scores is another popular mitigation deployed when releasing models, mainly by allowing the model to only output the class label (*18*). Figure 4B shows that this measure has a very small impact on our attack, only decreasing its accuracy from 96.0% when all digits are made available to 90.8% when only the label is on *n* = 3 variables (similarly, from 84.9 to 77.2% when *n* = 4 and from 78.4 to 71.9% when *n* = 5). We obtain similar results on MLP models, as shown on fig. S5.

DP is a popular privacy definition requiring that the model should not depend too strongly on any one record, as controlled by ϵ (*36*). DP protects individual privacy, and ML models trained with DP guarantees have been shown to prevent individual-level attacks, including MIAs. Although DP is not meant to protect population-level information such as correlations, the noise added might perturb the model enough to mitigate our attack. We train 1000 DP LR models with objective perturbation (*37*) using the Diffprivlib library (*38*) and different values of ϵ.

**Fig. 5.**
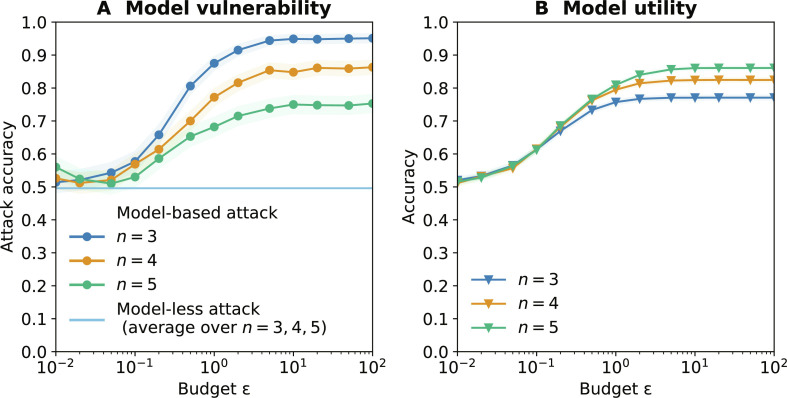
Trade-off between the vulnerability to our attack and the utility of DP Logistic Regression models. Trade-off between the vulnerability of DP LR models to our attack. (**A**) and their utility (**B**). We compute the metrics (with 95% confidence interval) over 1000 target models for *n* ∈ {3,4,5} variables.

Figure 5 shows that the vulnerability of a model to our attack is strongly linked to its utility, i.e., accuracy. For large value of ϵ, models are both useful and vulnerable. As we decrease ϵ, more noise is added, which reduces both the vulnerability of models to our attack and their utility. For small value of ϵ, our attack does not perform better than the model-less one, but at the cost of models losing any utility. Together, our results suggests these mitigations to be ineffective in preventing our attack,

#### Real-world dataset evaluation

We evaluate the performance of our attack on three real-world tabular datasets: Fifa19 (*39*), Communities and Crime (*40*), and Musk (*41*) (see section S6.2 for details). For each dataset, we select 100 data collections consisting of *n* = 4 columns, by sampling three different variables *X*_1_, *X*_2_, and *X*_3_ uniformly at random without replacement among the set of all triplets and then adding the output variable *Y*. We report the mean and SD of the attack accuracy averaged over 10 runs of 100 data collections each.

Contrary to the synthetic results above, the correlations in our real-world datasets are not uniform. Instead they are, to a different extent depending on the dataset, skewed toward zero. This means that a large fraction of the target correlations belong to the “low” bin [−1/3,1/3). Beyond this, it also means that the correlation constraints ρ(*X*_1_, *Y*) = cos θ_1_ and ρ(*X*_2_, *Y*) = cos θ_2_ are very small, thus leading to intervals *R* = [cos(θ_1_ + θ_2_), cos (θ_1_ − θ_2_)] that are close to but not equal to [−1,1] (e.g., *R* = [−0.99,0.99]). These intervals thus encompass the low bin fully and the other bins almost fully. For these two reasons, the model-less attack achieves on real-world datasets an “artificially” high accuracy. One solution would be to balance the test distribution. However, we instead prefer to show results for *N*_B_ = 5 bins. This reduces the artificial advantage of the model-less attack while maintaining the generality of the evaluation.

Table 1 shows that, despite its advantage, the model-less attack is outperformed by our model-based attack in five out of six cases. In Fifa19, it achieves an accuracy of 91.2% against LR models and 77.9% against MLP models, strongly outperforming the model-less attack that reaches 60.2%. Our attack similarly outperforms the model-less attack in the Communities and Crime dataset, achieving and accuracy of 86.0% against the LR models and 75.6% against the MLP models compared to that of 73.6% reached by the model-less attack. Last, while our attack achieves an accuracy of 82.0% on LR models, it is outperformed by the model-less attack when it comes to MLP models in the Musk dataset (56.3% versus 67.8%).

**Table 1.** Results of our CIAs on three real-world datasets. We abbreviate “Communities and Crime” to “C & C” for spacing reasons.

Number of bins	Dataset	Random guess	Model-less attack	Model-based attack	
				Logistic regression	MLP
*N*_B_ = 3	Fifa19	33.3	60.2 (5.0)	91.2 (3.6)	78.8 (4.5)
	C & C	33.3	73.6 (2.7)	86.0 (3.6)	75.6 (4.0)
	Musk	33.3	67.8 (5.6)	82.0 (3.2)	56.3 (6.2)
*N*_B_ = 5	Fifa19	20.0	29.4 (5.1)	79.1 (3.6)	61.2 (6.1)
	C & C	20.0	27.6 (3.2)	70.6 (3.8)	56.0 (5.3)
	Musk	20.0	28.7 (4.5)	72.0 (5.5)	41.7 (6.4)

However, as soon as we increase the number of bins, removing the artificial advantage of the model-less attack, our attack vastly and consistently outperforms the model-less attack. On Fifa19, it achieves an accuracy of 79.1% against LR models and 61.2% against MLP models, strongly outperforming the model-less attack that reaches 29.4%. It similarly outperforms the model-less attack on the Communities and Crime and Musk datasets. On Communities and Crime, it achieves an accuracy of 70.6% against the LR models and of 56.0% against the MLP models compared to that of 27.6% reached by the model-less attack. On Musk, it achieves 72.0% against the LR models and 41.7% against the MLP models compared to the 28.7% reached by the model-less attack.

### Attribute inference attack

We have, so far, shown that dataset correlations, which are fundamental summary statistics about a dataset, can be extracted from an ML model trained on it. We here argue that the ability of an attacker to accurately extract statistical information can enable other attacks, particularly allowing weaker attackers to infer membership or attributes from trained ML models. More specifically, we present an AIA that uses as building blocks the correlations between input variables extracted from the model using our attack.

We assume the first variable in the dataset *X*_1_ to be sensitive and consider an adversary that aims to recover the sensitive attribute value *x*_1_ of a target record (*x*_1_, *x*_2_, …, *x*_*n*−1_, *y*) ∈ *D*_T_ using black-box access to a model M_T_ trained on *D*_T_. In line with the literature (*14*, *42*, *43*), we assume that the adversary knows the partial record (*x*_2_, …, *x*_*n*−1_), the label *y*, and the one-way marginals of the attributes.

The intuition behind our attack is that the correlation between the sensitive variable *X*_1_ and the other variables, when combined with knowledge of the partial record (*x*_2_, …, *x*_*n*−1_, *y*), can lead to accurate inference of the sensitive attribute *x*_1_. We propose an attack that extracts the correlations between the input variables ρ(*X_i_*, *X_j_*),1 ≤ *i* < *j* ≤ *n* − 1 from the model using our model-based attack, under default scenario S2 and uses them to generate synthetic data satisfying these correlations. We then search for (approximate) matches of the partial record (*x*_2_, …, *x*_*n*−1_, *y*) in the synthetic data and return as prediction ${\hat{x}}_{1}$ the average value of the sensitive attribute values of matched records. We refer the reader to Attribute inference attack methodology for the details.

We evaluate our correlation inference–based AIA (CI-AIA) on the Fifa19 dataset (*39*), randomly selecting 1000 data collections consisting of *n* = 4 columns. For each data collection, we train an LR model and run the attack against 500 randomly selected records (see section S9 for the details). To evaluate the attack performance, we divide the range of the sensitive attribute *X*_1_ into three bins corresponding to the tertiles of the distribution. Thus, a majority baseline attains an accuracy of roughly 33%.

We compare our attack with five attacks from previous works: the first AIA by Fredrikson *et al.* (*14*), confidence score-based model inversion attack (CSMIA) by Mehnaz *et al.* (*43*), the threshold-based attack by Yeom *et al.* (*24*), and the confidence-based attribute inference (CAI) and weighted CAI (WCAI) methods of Jayaraman and Evans (*42*). These methods are described in section S9. We further compare our approach with two other baselines: a naïve approach that returns a random sample from the one-way marginal of the sensitive attribute *X*_1_, which is known to the adversary; and another approach, referred to as *X*_1_ − *Y* correlation, that runs our CI-AIA approach on the variables *X*_1_ and *Y* alone. Its goal is to quantify the leakage from the adversary knowledge of ρ(*X*_1_, *Y*) without access to the model. Knowledge of the correlation ρ(*X*_1_, *Y*) can lead to better than random inference of the attribute *x*_1_ because *y* is known. This baseline acts as a lower bound for CI-AIA, as the latter additionally assumes access to the model.

Table 2 shows that our CI-AIA outperforms previous works and baselines, demonstrating how correlations can be used as building blocks for other attacks. Our attack achieves an accuracy of 49.7%, significantly better than CSMIA [by Mehnaz *et al.* (*43*)], which only reaches 46.5% and is on par with CAI [by Jayaraman and Evans (*42*)], and the method by Fredrikson *et al.* (*14*), which reaches 38.4% and is on par with that by Yeom *et al.* (*24*). Our attack also outperforms the *X*_1_ − *Y* correlation baseline, confirming our intuition that the additional correlations ρ(*X*_1_, *X_i_*), *i* > 1 extracted from the model using our attack lead to more accurate attribute inference.

**Table 2.** Comparison between CI-AIA and other AIAs on Fifa19. We report the attack accuracy averaged over 1000 runs (with 95% confidence interval). The best performing method is highlighted in bold.

Method	Accuracy
(Ours) CI-AIA	**49.7 ± 1.0**
Fredrikson *et al.* (*14*)	38.4 ± 0.8
CSMIA [Mehnaz *et al.* (*43*)]	46.5 ± 0.9
Yeom *et al.* (*24*)	38.5 ± 0.8
CAI [Jayaraman and Evans (*42*)]	46.5 ± 0.9
WCAI [Jayaraman and Evans (*42*)]	41.7 ± 0.8
*X*_1_ − *Y* correlation	41.7 ± 0.8
Marginal prior	35.3 ± 0.6

## DISCUSSION

In this work, we study a previously unknown type of leakage in ML models, the leakage of correlations between input variables, proposing the first CIA against ML models. Our results show that a model remembers more information than previously thought, raising privacy and confidentiality concerns whenever dataset correlations are sensitive information. We develop two different attacks, model-less and model-based, that together allow to correctly quantify the leakage from the model. We perform an extensive evaluation of our attacks, showing that models leak correlations of their training dataset with high accuracy, in both synthetic and real-world datasets. We also show that mitigations such as limiting the number of queries that can be made to the model, their precision, or training the models with DP guarantees do not help. Last, we show how correlations extracted using our attack can be used as building blocks for AIAs, particularly enabling weak adversaries to develop more powerful attacks than previously thought.

Attack motivation: To further emphasize the privacy interest of correlation information, we here describe several use cases where correlation leakage might represent a privacy violation or could lead to individual harm. First, we consider the example presented in the introduction: a scoring model for depression that leaks that patients living in the inner city are more likely to have used illegal substances, an information that could be used to target them. Second, we consider a model controlling the signalization on the highway. We assume that it was trained on data collected from vehicles in a specific area and that there is a positive correlation between the darkness of the car and the probability of accident in the area. If the model leaks this information to an insurance company operating in the area, then this could lead to higher insurance premium for darker cars in the area. Third, we consider a model that leaks the correlation between age and income of people in its training dataset. Furthermore, assume that the model developer claims the model to be trained on representative data. Our CIA can be used as part of an auditing process, to surface a situation where, although the training dataset is balanced along each individual attribute (e.g., age and income taken in isolation), it is not balanced on their intersection, e.g., in the training dataset being older is excessively correlated with income.

When does the leakage happen? Our results (Fig. 2 and fig. S7) suggest that (i) models leak the correlation between input variables more when these variables are highly correlated (positively or negatively) with the output variable *Y* than when these variables are not correlated with *Y*, but that (ii) leakage still occurs even when there is little to no correlation between these variables and the target variable. The second finding might seem particularly unexpected in the case of *n* = 3 variables, where the resulting model makes random predictions because all its input variables (*X*_1_ and *X*_2_) are statistically independent to the target variable *Y* (uncorrelation implies statistical independence for Gaussian variables). The apparent contradiction can, however, be explained by the fact that a model making random predictions is not the same as the model not having any statistical dependency on its training dataset. While the optimal model is only intended to learn *P*(*Y* ∣ *X* = *x*), current learning techniques such as stochastic gradient descent optimize the expected loss computed over records of the joint distribution *P*(*X*, *Y*). Thus, the distribution of the input variables *P*(*X*) influences the model. Whether the leakage that we observe is a necessary condition for learning to happen and thus whether robust defenses might exist remain an open problem. We now analyze some of the assumptions made on the adversary knowledge.

Access to auxiliary data: Our approach does not require access to the private dataset *D*_T_ or to a dataset drawn from a similar distribution. Such detailed knowledge of the data is a common assumption in the literature on inference attacks and has led to strong results as the adversary uncertainty is reduced, e.g., (*17*, *26*, *27*). We, however, believe that real-world adversaries are unlikely to have detailed knowledge of the data. Our goal is thus to explicitly extract information about the data distribution and use it to infer attributes of individual records. While, in this work, we have assumed a Gaussian copula prior on the underlying distribution (allowing us to control for correlations), future work could explore different priors to improve our results.

**Fig. 6.**
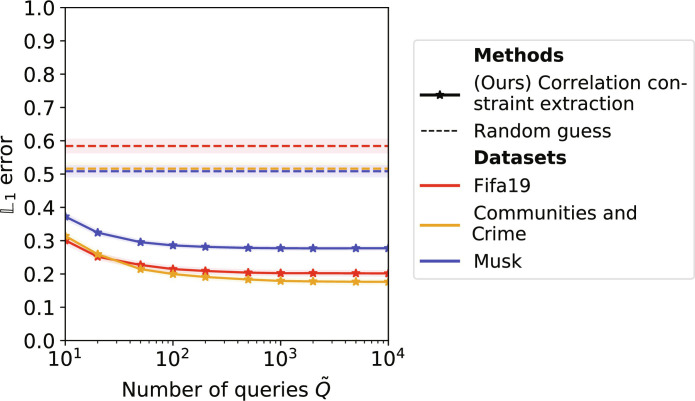
Correlation constraint extraction from LR models for different number of queries $\tilde{Q}$. We compute the $L_{1}$ error between the inferred and the ground-truth values of the correlations: $\frac{1}{n-1}\sum_{i=1}^{n-1}‍|\hat{\rho }\left(X_{i},Y\right)-\rho \left(X_{i},Y\right)|$ (mean with 95% confidence interval).

Knowledge of the correlation constraints: Our default scenario S2 assumes the adversary to know [ρ(*X_i_*, *Y*)]_*i*=1,…,*n*−1_. We show that these can be extracted from the model using a small number of queries $\tilde{Q}$ . More specifically, we sample $\tilde{Q}$ records (*x*_1_, …, *x*_*n*−1_) independently from the marginals *F*_1_, …, *F*_*n*−1_ and then query the model to retrieve the predicted label *y*. We then estimate ${\left[\hat{\;\rho }\left(X_{i},Y\right)\right]}_{i=1,\ldots ,n-1}$ on these records. We compute the $L_{1}$ error of the predictions, defined as $\frac{1}{n-1}\sum_{i=1}^{n-1}‍|\hat{\rho }\left(X_{i},Y\right)-\rho \left(X_{i},Y\right)|$ , over 500 data collections of *n* = 4 columns, and compare it with the $L_{1}$ error of a random guess in [−1,1]. Figure 6 shows our approach to perform much better than the random guess, reducing the $L_{1}$ error from 0.584 to 0.214 with as little as 100 queries to LR models trained on the Fifa19 dataset. The performance of our approach improves as the number of queries $\tilde{Q}$ increases but stabilizes early at $\tilde{Q}=100$.

Knowledge of precise marginals: We have so far assumed the adversary to have precise knowledge of the marginals, discretizing them for simplicity into *G* = 100 subintervals of equal size. Figure S13 shows that decreasing *G* and, by doing so, reducing the adversary’s ability to sample from the marginals with fine granularity have a very small impact on the performance of our attack. The accuracy on Fifa19 decreases very slowly from 91.2% (*G* = 100) to 82.9% (*G* = 5), much higher than the model-less attack at 60.2%.

**Fig. 7.**
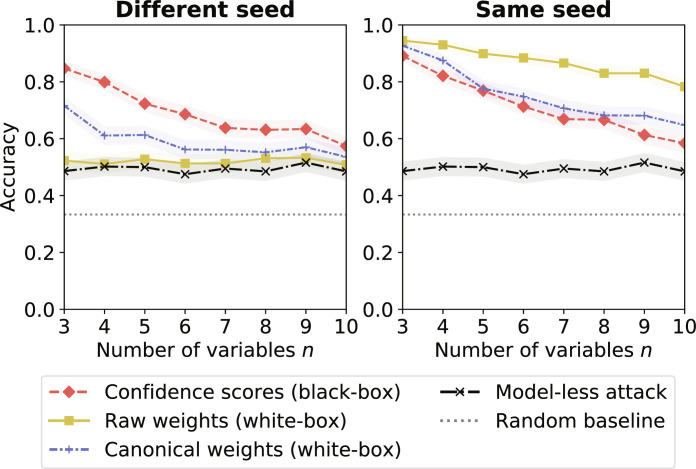
Impact of the adversary’s knowledge of the model seed on CIA performance against MLPs. We compare black-box and white-box CIAs against MLPs when the adversary uses a different seed (**left**) or the same seed (**right**) as the target model for training each model used to generate the meta-classifier’s training dataset. We report the mean attack accuracy (with 95% confidence interval).

Knowledge of the model seed: We believe that it is more realistic to assume that the adversary does not know the seed used to initialize the training algorithm of MLP models, including their weights. This assumption leads to more uncertainty, as the models may end up in different local minima compared to the target model and may behave less similarly than expected. This is not an issue for LR models, as they minimize a convex objective having a global optimum (*11*). Figure 7 (left and right) compares the performances of black-box and various white-box CIAs against MLP models, when changing, respectively keeping the seed constant between models used to generate the meta-classifier’s training dataset and target models. We refer the reader to section S7 for implementation details of the white-box attacks. While the black-box attack gains little from the seed knowledge [up to 4.6 percentage points (p.p.)], the white-box attack reaches an astonishing 94.5% for *n* = 3 variables, 88.4 for *n* = 6, and 78.3% for *n* = 10 variables, the last being 29.8 p.p. above the model-less attack. It now also outperforms the attack on LR models for all *n* ≥ 4, confirming our hypothesis that more weights (e.g., 432 for the MLP versus 10 for the LR when *n* = 10) can encode more information. Last, the canonical sorting of weights, proposed by Ganju *et al.* (*26*), to enhance the performance of the meta-classifier is, in this setting, somewhat effective when the seed changes. However, its performance is still much lower than the black-box attack. It, however, achieves the opposite effect of destroying some of the information present in the raw weights when the seed is kept constant.

## MATERIALS AND METHODS

In this section, we describe in detail our methodology for our CIA (see Correlation inference attack) and our CI-AIA (see Attribute inference attack methodology).

### Correlation inference attack

We describe in detail the various building blocks of our methodology for the model-less and model-based attacks. More specifically, we first describe our algorithms for generating correlation matrices under constraints (see the “Generating correlation matrices under constraints” section). Second, we describe how we generate synthetic dataset conditionally on a correlation matrix for arbitrary one-way marginals (see the “Generating synthetic data given a correlation matrix for arbitrary one-way marginals” section). Third, we describe the details of our model-based attack (see the “Model-based attack” section).

#### Generating correlation matrices under constraints

We adapt the algorithm of Numpacharoen and Atsawarungruangkit (*32*), which we include in section S3 (algorithm S1), to our problem of generating matrices under constraints given by the adversary’s knowledge. We notice that the coefficients of the first column of *B* (cos θ_*i*,1_ for *i* = 2, …, *n* − 1) are identical to those of *C* and are free parameters. We start by reordering the variables to *Y*, *X*_1_, …, *X*_*n*−1_ to generate ρ(*X*_1_, *X*_2_) first (and explain below why). Under S2, we set the coefficients of the first column of *B* (and *C*) equal to the correlation constraints [ρ(*X_i_*, *Y*)]_*i*=1,…,*n*−1_. This amounts to replacing line 6 from algorithm S1 (highlighted in red) with *c*_*i*,1_ ← ρ(*X*_*i*−1_, *Y*). Under S1, we will set the first two coefficients equal to the correlation constraints ρ(*X*_1_, *Y*) and ρ(*X*_2_, *Y*), initializing the others uniformly at random within [−1,1]. We replace line 6 from algorithm S1 correspondingly.

We then shuffle the variables 3 to *n* − 1 by applying a random permutation. Naïvely applying algorithm S1 would generate the correlations using the default variable ordering (in our case, *Y*, *X*_1_, …, *X*_*n*−1_). While the correlation generated first, ρ(*X*_1_, *X*_2_), is uniformly distributed within its boundaries, the correlation generated last, ρ(*X*_*n*−2_, *X*_*n*−1_), is not, because the previously sampled correlations restrict its range. More specifically, its conditional distribution follows a bell-shaped curve. Shuffling the variables reduces this bias (*32*). We choose to keep the target pair *X*_1_ and *X*_2_ in the first position to guarantee a uniform prior over the distribution of the target correlation ρ(*X*_1_, *X*_2_). We assume that the adversary does not know how ρ(*X*_1_, *X*_2_) is distributed within its boundaries. Furthermore, we do not want to bias the values toward the center of the interval, as would be the case if the value was not generated first. Algorithm 1 details how we carefully sequence the shuffling, the column reordering, and the inversion of the shuffling for scenario S2 (see algorithm S2 in section S4 for scenario S1).


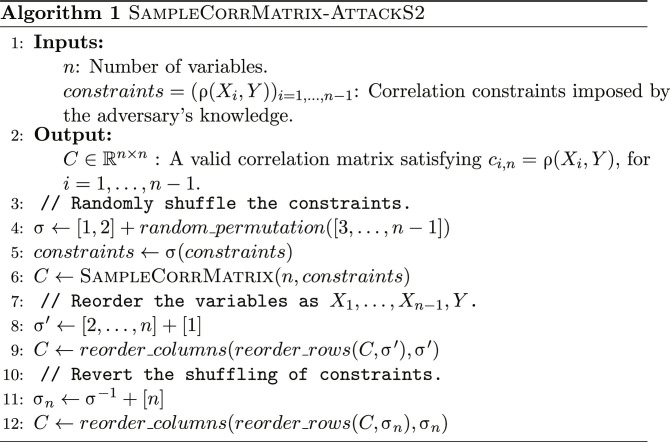


Algorithm 2 details our procedure to sample the unknown value ρ(*X*_1_, *X*_2_) for scenario S3, when all the other correlations are known. We start by placing the target pair (*X*_1_, *X*_2_) in the last position. Denote by *C*′ the partial correlation matrix after reordering the variables (filling the unknown *c*′_*n*,*n*−1_ with a dummy value). We sample the last coefficient *c*′_*n*,*n*−1_ uniformly within its bounds, which we determine by reverse-engineering part of the spherical parameterization *B*. More specifically, we compute all the low triangular coefficients except for *b*_*n*,*n*−1_ and *b*_*n*,*n*_, which cannot be determined absent knowledge of *c*′_*n*,*n*−1_. This allows us to compute the bounds of *c*′_*n*,*n*−1_ and sample it uniformly within them.


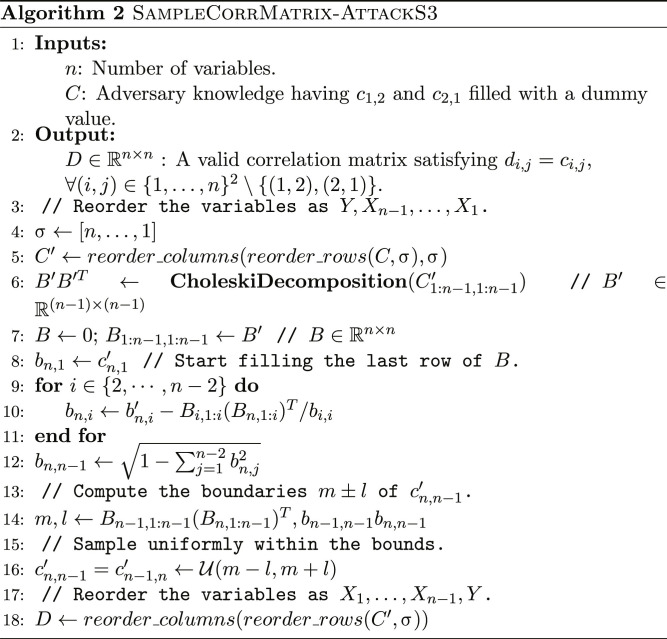


We discuss in section S3 an alternative, optimization-based approach to generate correlation matrices under constraints, that we tried in preliminary experiments and we found to be ineffective.

#### Generating synthetic data given a correlation matrix for arbitrary one-way marginals

Algorithm 3 details our heuristic to modify the correlation constraints. More specifically, it determines *V*′ such that, on average, synthetic datasets *D* generated from matrices matching *V*′ minimize the difference max_(*i*,*j*)∈*P*_ ∣ *E*[*C*(*D*)_*i*,*j*_] − (*c*_T_)_*i*,*j*_∣ between their empirical correlations *C*(*D*) and the correlation constraints. For conciseness, we focus on the default scenario S2, where *V* = [ρ(*X_i_*, *Y*)]_*i*=1,…,*n*−1_ (of which scenario S1 is a particular case).

#### Model-based attack

Our model-based attack exploits access to an ML model M_T_ trained on the target dataset *D_T_* to extract the target correlation. First, we generate *K* correlation matrices *C*^1^, …, *C^K^* satisfying *V* when the marginals are standard normals (and *V*′ for arbitrary marginals). In each scenario, the correlation matrices are sampled using the corresponding algorithm. For each correlation matrix *C^k^*, we generate a synthetic dataset *D^k^* whose correlations approximately match *V* and whose marginals match *F_i_*, *i* = 1, …, *n* as described in the “Generating synthetic data given a correlation matrix for arbitrary one-way marginals” section.

Second, we train a model M*^k^* on dataset *D^k^*, for every *k* = 1, …, *K*. The models are trained using the same algorithm and hyperparameters as the target model but a different seed. We present in Discussion the results of our attack when the adversary knows the seed.

Third, we generate a synthetic query dataset *D*_query_ in the same way as above and use it to extract black-box features from the models. More specifically, after the *K* models have been trained, we pass the records of *D*_query_ through the model M*^k^* for *k* = 1, …, *K* and retrieve the confidence scores for the first *L* − 1 classes (the last one is redundant with the others as they all sum to 1). Denoting by *Q* the size of *D*_query_, we thus extract a total of *Q* × (*L* − 1) features from each model. Here, we assume for simplicity that the models are binary classifiers (*L* = 2), meaning that we extract *Q* features from each model. We concatenate them to form the output feature vectors *O_k_*, *l* = 1, …, *K*. Our hypothesis is that these features are likely to encode information about the dataset correlations, e.g., that a model makes more confident predictions on records resembling its own training dataset.

Fourth, given a dataset *D*, we denote by *C*(*D*) the correlation matrix computed on the dataset. We label each feature vector *O_k_* with the correlation *L_k_* = *C*(*D^k^*)_1,2_ of the corresponding synthetic dataset, discretized over *B* classes. We then train a meta-classifier A: ℝ*^Q^* → {1, …, *B*} on *D*_meta_ = {(*O_k_*, *L_k_*), *k* = 1, …, *K*} to infer the correlation given features extracted from the model.

Last, we deploy the meta-classifier on the target model M*_T_*. The adversary’s guess is the output class A(*O*_T_) predicted by the meta-classifier on the feature vector *O*_T_, consisting of the outputs of M_T_ on the query dataset *D*_query_.

### Attribute inference attack methodology

We describe our AIA aiming to infer the sensitive attribute value *x*_1_ of a target record (*x*_1_, …, *x*_*n*−1_, *y*) ∈ *D*_T_ using black-box access to a model M_T_ trained on a private dataset *D_T_*. Our method consists of three steps.

Step 1: We execute our CIA independently against each target pair (*X_i_*, *X_j_*),1 ≤ *i* < *j* ≤ *n* − 1 under default scenario S2. The output of the attack is a correlation bin *b*_*i*, *j*_ ∈ {1, …, *N*_B_}. To evaluate our attack on real-world datasets having arbitrary marginals, we run the attack using the ModifyCorrelationConstraints heuristic. We denote by *V*′_*i*, *j*_ ∈ ℝ^*n*−1^ the modified correlation constraints returned by the heuristic.

Step 2: We sample synthetic datasets whose empirical correlations belong to the inferred bins while also matching the correlation constraints [ρ(*X_i_*, *Y*)]_*i*=1,…,*n*−1_. Sampling synthetic data under constraints given by the adversary knowledge requires to modify the correlation constraints ρ(*X_i_*, *Y*), *i* = 1, …, *n* − 1, as described in the “Generating synthetic data given a correlation matrix for arbitrary one-way marginals” section. We here use the average of modified constraints used previously to attack each of the pairs: $V\prime =\frac{2}{\left(n-1\right)\left(n-2\right)}\sum_{1\leq i<j\leq n-1}‍V\prime_{i,j}$ . We generate *S*′ synthetic datasets under these modified constraints and then select among them the datasets *D* whose empirical correlations *C*(*D*)_*i*,*j*_ belong to the inferred bin *b*_*i*,*j*_, for every 1 ≤ *i* < *j* ≤ *n* − 1, discarding the rest. We concatenate the datasets together into a larger dataset *D*_synth_.

Step 3: We select all the values of the sensitive attribute $x_{1}^{1},\ldots ,x_{1}^{H}$ belonging to records that approximately match the partial record (*x*_2_, …, *x*_*n*−1_, *y*). We search for approximate matches as follows. First, we discretize the marginals of nonsensitive variables *X*_2_, …, *X*_*n*−1_ into *G* subintervals of same size and denote by *b*_2_, …, *b*_*n*−1_ the size of the subintervals. Second, we retrieve all the records (*x*_1′_, …, *x*_*n*−1_′, *y*′) in *D*_synth_ such that (i) ∣*x_i_* − *x*′*_i_* ∣ ≤ *m_i_b_i_*, *i* = 2, …, *n* − 1 and (ii) *y* = *y*′, where *m_i_* denotes the resolution of the search around the partial record’s attribute *x_i_*. For instance, for *m*_2_ = 1 and *b*_2_ = 0.2, we only select records having *x*′_2_ ∈ [*x*_2_ − 0.2, *x*_2_ + 0.2]. If no record is found, then we increase the resolution parameters by increments δ*_i_*: *m_i_* ← *m_i_* + δ*_i_*, *i* = 2, …, *n* − 1, until at least one record is found. We return the average sensitive attribute of matched records ${\hat{x}}_{1}=\frac{1}{H}\left(\sum_{h=1}^{H}‍x_{1}^{h}\right)$ as our prediction. We also explore ablations of our attack using different formulas for predicting *x*_1_ given the partial record (*x*_2_, …, *x*_*n*−1_, *y*) and the synthetic data generated under step 2, including variations of Jayaraman and Evans’s (*42*) formula, none of which significantly improve the attack’s performance. Summary of the results can be found in section S9. As for the *X*_1_ − *Y* correlation baseline, we run steps 2 and 3 of our CI-AIA approach, described above, on variables *X*_1_ and *Y*, but not step 1 as there is no need to extract additional correlations from the model.

## RELATED WORK

Information leakage by ML models has, so far, been mostly studied through the lens of individual records. This includes MIAs (*15*, *17*, *18*, *21*, *22*, *24*) aiming to infer if a record was used to train a model—and, to a lesser extent, attribute inference (*13*, *14*, *43*) and reconstruction (*11*, *44*) attacks—aiming to reconstruct respectively, partially and entirely, a record. While potentially very invasive from a privacy perspective, attacks against individual-level records commonly rely on powerful adversaries, with partial (*21*) or near-complete (*11*) knowledge of the dataset or with access to a dataset sampled from the same distribution. We here focus on an adversary aiming to infer correlations without access to real records, which requires rethinking the current design of inference attacks. In particular, we develop a model-less attack as a strong baseline and use Gaussian copulas to sample synthetic tabular datasets that we use to generate the meta-classifier’s training dataset.

PIAs study the leakage of macro properties of the dataset. Ateniese *et al.* (*10*) introduced PIAs, albeit by another name, and applied them to hidden Markov models and support vector machines. They show, for instance, that it is possible to distinguish the dialect on which a speech recognition model was trained. To this end, they train a meta-classifier on white-box features extracted from models trained in the same way as the target model, a methodology now known as shadow modeling (*15*). Ganju *et al.* (*26*) showed that extending this attack to MLPs is more challenging due to their permutation equivalence property and propose to use weight-based neuron sorting and set-based meta-classifiers; we use the former in our white-box experiments on MLPs. Zhang *et al.* (*27*) proposed the first black-box PIA, making use of the model’s output probabilities computed on a dataset of real records from the same distribution. Last, Suri and Evans (*45*) proposed a formal definition of distribution inference attacks that unifies the PIAs of previous works, which focused on the leakage of the proportion of samples along a certain attribute. The authors extend the definition to other properties such as the average node degree of a graph.

Our work differs substantially from previous work in PIAs. First, we focus, by design, on explicit micro characteristics of the dataset *X* rather than on external macro properties of the record (e.g., the gender label of an image). Second, we demonstrate how dataset-level information, here correlations, can be used as building blocks for more accurate AIAs. Third, our attack, contrary to PIAs, does not require access to a subset of the dataset or, at least, a dataset sampled from a similar distribution. This would mean that the adversary could readily compute the correlations or, at least, have a very good prior on them. We here do not assume access to private data for approximating the distribution, developing a methodology for inferring correlations, which relies on synthetic data instead of private data. More specifically, the models that we use to generate the training dataset of the meta-classifier are trained exclusively on synthetic records. This makes the attack accessible to weaker adversaries than those of previous works. Fourth, previous works mainly focus on a categorical choice between very distinct properties. For instance, Ganju *et al.* (*26*) aim to infer whether the proportion of older faces is equal to (precisely) 23 or 37% or whether exactly 0 or 87% of the faces were from white people (*26*). By contrast, we frame the correlation inference task as a prediction task covering the entire range of possible values. While Zhou *et al.* (*46*) aim to address this shortcoming by framing property inference as a regression task, they only apply their attack to generative adversarial networks, which would, by design, reflect the statistical properties of the training dataset making a correlation attack trivial.

AIAs aim to recover the sensitive attribute of a target record. Friedrikson *et al.* (*14*) proposed the first AIA against ML models. They applied their attack to linear regression models available as a black-box and aimed to infer a patient’s genotype. Subsequent work (*13*) extended this attack to make use of confidence scores and applied it to decision trees. Yeom *et al.* (*24*) explored the connection between membership and attribute inference and proposed a black-box attack relying on a membership inference oracle. Mehnaz *et al.* (*43*) then proposed two black-box attacks: (i) a confidence-based attack dropping the prior work’s assumption that the adversary knows the one-way marginals of the dataset and (ii) a label-based attack assuming the adversary has access to a large fraction of the training dataset (excluding the sensitive attribute).

In contemporaneous work, Jayaraman and Evans (*42*) propose two extensions of the black-box AIA of Yeom *et al.* (*24*) and a white-box AIA. Our black-box CI-AIA is fundamentally different from their black-box AIAs, because we study a weaker adversary who does not know the data distribution via access to auxiliary data. Instead, we (1) extract information about the distribution from the model (the correlation between the input variables), (2) use this information to generate synthetic data, and (3) predict the sensitive attribute using the synthetic data. Table 2 shows our CI-AIA to outperform their black-box AIAs when compared fairly in our weak adversary setting. We further note that our step (3) applied to synthetic data bears similarity with Jayaraman and Evans’s (*42*) prediction rule applied to auxiliary data. To understand whether their prediction rule can further improve our CI-AIA performance, we substituted their prediction rule to step (3) of our attack while leaving our steps (1) and (2) unchanged. We show in section S9 that this does not lead to better performance than our CI-AIA.

Synthetic data–based shadow modeling is an under-explored research area. Shokri *et al.* (*15*) were the first to use synthetic data to train shadow models. They developed a hill-climbing algorithm to generate synthetic records. Their approach is based on the intuition that a model would be more confident on records that are similar to the training dataset. Salem *et al.* (*22*) argued that this method is only efficient for datasets of binary records and explored the use of shadow datasets from different sources. This is, however, not a suitable option when aiming to infer summary statistics of a dataset as it requires shadow datasets to span the entire range of possible values for the statistics.

## Supplementary Materials

### This PDF file includes:

Sections S1 to S9 Figs. S1 to S9 Table S1 Algorithm S1 to S3 References

## References

[1] S. Qummar, F. G. Khan, S. Shah, A. Khan, S. Shamshirband, Z. U. Rehman, I. A. Khan, W. Jadoon, A deep learning ensemble approach for diabetic retinopathy detection. IEEE Access 7, 150530–150539 (2019).

[2] Y. Wu, M. Schuster, Z. Chen, Q. V. Le, M. Norouzi, W. Macherey, M. Krikun, Y. Cao, Q. Gao, K. Macherey, J. Klingner, A. Shah, M. Johnson, X. Liu, Ł. Kaiser, S. Gouws, Y. Kato, T. Kudo, H. Kazawa, K. Stevens, G. Kurian, N. Patil, W. Wang, C. Young, J. Smith, J. Riesa, A. Rudnick, O. Vinyals, G. Corrado, M. Hughes, J. Dean, Google’s neural machine translation system: Bridging the gap between human and machine translation. arXiv:1609.08144 (2016).

[3] Siri Team (Apple), Deep learning for siri’s voice: On-device deep mixture density networks for hybrid unit selection synthesis (2017); https://machinelearning.apple.com/research/siri-voices.

[4] Amazon Rekognition, Moderating content; https://docs.aws. amazon.com/rekognition/latest/dg/moderation.html.

[5] H. Chen, O. Engkvist, Y. Wang, M. Olivecrona, T. Blaschke, The rise of deep learning in drug discovery. Drug Discov. Today 23, 1241–1250 (2018). 29366762 10.1016/j.drudis.2018.01.039

[6] A. Gordo, J. Almazan, J. Revaud, D. Larlus, “Deep image retrieval: Learning global representations for image search” in *Computer Vision–ECCV 2016: 14th European Conference, Amsterdam, The Netherlands, October 11–14, 2016, Proceedings, Part VI 14* (Springer, 2016), pp. 241–257.

[7] I. Goodfellow, Y. Bengio, A. Courville, *Deep Learning* (MIT Press, 2016).

[8] A. Alvi, P. Kharya, Using deepspeed and megatron to train megatron-turing nlg 530b, the world’s largest and most powerful generative language model (2021); www.microsoft.com/en-us/research/blog/using-deepspeed-and-megatron-to-train-megatron-turing-nlg-530b-the-worlds-largest-and-most-powerful-generative-language-model/.

[9] M. Veale, R. Binns, L. Edwards, Algorithms that remember: Model inversion attacks and data protection law. Philos. Trans. R. Soc. A Math. Phys. Eng. Sci. 376, 20180083 (2018). 10.1098/rsta.2018.0083 PMC6191664 30322998

[10] G. Ateniese, L. V. Mancini, A. Spognardi, A. Villani, D. Vitali, G. Felici, Hacking smart machines with smarter ones: How to extract meaningful data from machine learning classifiers. Int. J. Secur. Netw. 10, 137–150 (2015).

[11] B. Balle, G. Cherubin, J. Hayes, “Reconstructing training data with informed adversaries” in *2022 IEEE Symposium on Security and Privacy (SP)* (IEEE, 2022), pp. 1138–1156.

[12] N. Carlini, F. Tramèr, E. Wallace, M. Jagielski, A. Herbert-Voss, K. Lee, A. Roberts, T. Brown, D. Song, Ú. Erlingsson, A. Oprea, C. Raffel, “Extracting training data from large language models” in *USENIX Security Symposium*, vol. 6 (USENIX, 2021).

[13] M. Fredrikson, S. Jha, T. Ristenpart, “Model inversion attacks that exploit confidence information and basic countermeasures” in *Proceedings of the 22nd ACM SIGSAC Conference on Computer and Communications Security* (ACM, 2015), pp. 1322–1333.

[14] M. Fredrikson, E. Lantz, S. Jha, S. Lin, D. Page, T. Ristenpart, “Privacy in pharmacogenetics: An end-to-end case study of personalized warfarin dosing” in *23rd USENIX Security Symposium (USENIX Security 14)* (USENIX, 2014), pp. 17–32. PMC4827719 27077138

[15] R. Shokri, M. Stronati, C. Song, V. Shmatikov, “Membership inference attacks against machine learning models” in *2017 IEEE Symposium on Security and Privacy (SP)* (IEEE, 2017), pp. 3–18.

[16] N. Homer, S. Szelinger, M. Redman, D. Duggan, W. Tembe, J. Muehling, J. V. Pearson, D. A. Stephan, S. F. Nelson, D. W. Craig, Resolving individuals contributing trace amounts of DNA to highly complex mixtures using high-density SNP genotyping microarrays. PLOS Genet. 4, e1000167 (2008). 18769715 10.1371/journal.pgen.1000167 PMC2516199

[17] N. Carlini, S. Chien, M. Nasr, S. Song, A. Terzis, F. Tramer, “Membership inference attacks from first principles” in *2022 IEEE Symposium on Security and Privacy (SP)* (IEEE, 2022), pp. 1897–1914.

[18] C. A. Choquette-Choo, F. Tramer, N. Carlini, N. Papernot, “Label-only membership inference attacks” in *International Conference on Machine Learning* (PMLR, 2021), pp. 1964–1974.

[19] B. Jayaraman, D. Evans, “Evaluating differentially private machine learning in practice” in *28th USENIX Security Symposium (USENIX Security 19)*, (USENIX, 2019), pp. 1895–1912.

[20] K. Leino, M. Fredrikson, “Stolen memories: Leveraging model memorization for calibrated white-box membership inference” in *29th USENIX security symposium (USENIX Security 20)* (USENIX, 2020), pp. 1605–1622.

[21] M. Nasr, R. Shokri, A. Houmansadr, “Comprehensive privacy analysis of deep learning: Passive and active white-box inference attacks against centralized and federated learning” in *2019 IEEE Symposium on Security and Privacy (SP)* (IEEE, 2019), pp. 739–753.

[22] A. Salem, Y. Zhang, M. Humbert, P. Berrang, M. Fritz, M. Backes, Ml-leaks: Model and data independent membership inference attacks and defenses on machine learning models. arXiv:1806.01246 (2018).

[23] S. Truex, L. Liu, M. E. Gursoy, L. Yu, W. Wei, Demystifying membership inference attacks in machine learning as a service. IEEE Trans. Serv. Comput. 14, 2073–2089 (2021).

[24] S. Yeom, I. Giacomelli, M. Fredrikson, S. Jha, “Privacy risk in machine learning: Analyzing the connection to overfitting” in *2018 IEEE 31st Computer Security Foundations Symposium (CSF)* (IEEE, 2018), pp. 268–282.

[25] J. Buolamwini, T. Gebru, “Gender shades: Intersectional accuracy disparities in commercial gender classification” in *Conference on Fairness, Accountability and Transparency* (PMLR, 2018), pp. 77–91.

[26] K. Ganju, Q. Wang, W. Yang, C. A. Gunter, N. Borisov, “Property inference attacks on fully connected neural networks using permutation invariant representations” in *Proceedings of the 2018 ACM SIGSAC Conference on Computer and Communications Security* (ACM, 2018), pp. 619–633.

[27] W. Zhang, S. Tople, O. Ohrimenko, “Leakage of dataset properties in multi-party machine learning” in *USENIX Security Symposium* (USENIX, 2021), pp. 2687–2704.

[28] P. Waldmann, On the use of the pearson correlation coefficient for model evaluation in genome-wide prediction. Front. Genet. 10, 899 (2019). 31632436 10.3389/fgene.2019.00899 PMC6781837

[29] H. Zhou, Z. Deng, Y. Xia, M. Fu, A new sampling method in particle filter based on pearson correlation coefficient. Neurocomputing 216, 208–215 (2016).

[30] European Commission, “On artificial intelligence - a european approach to excellence and trust” (European Commission, 2020); https://ec.europa.eu/info/sites/default/files/commission-white-paper-artificial-intelligence-feb2020_en.pdf.

[31] J. C. Pinheiro, D. M. Bates, Unconstrained parametrizations for variance-covariance matrices. Stat. Comput. 6, 289–296 (1996).

[32] K. Numpacharoen, A. Atsawarungruangkit, Generating correlation matrices based on the boundaries of their coefficients. PLOS ONE 7, e48902 (2012). 23152816 10.1371/journal.pone.0048902 PMC3495965

[33] M. Sklar, “Fonctions de répartition à n dimensions et leurs marges”, in *Annales de l’ISUP*, vol. 8 (HAL Open Science, 1959), pp. 229–231.

[34] Q. Xiao, Calculating correlation coefficient for gaussian copula. arXiv:1608.00738 (2016).

[35] A. Choromanska, M. Henaff, M. Mathieu, G. B. Arous, Y. LeCun, “The loss surfaces of multilayer networks” in *Artificial Intelligence and Statistics* (PMLR, 2015), pp. 192–204.

[36] C. Dwork, F. McSherry, K. Nissim, A. Smith, “Calibrating noise to sensitivity in private data analysis” in *Theory of Cryptography: Third Theory of Cryptography Conference, TCC 2006, New York, NY, USA, March 4–7, 2006. Proceedings 3* (Springer, 2006), pp. 265–284.

[37] K. Chaudhuri, C. Monteleoni, A. D. Sarwate, Differentially private empirical risk minimization. J. Mach. Learn. Res. 12, 1069–1109 (2011). 21892342 PMC3164588

[38] N. Holohan, S. Braghin, P. M. Aonghusa, K. Levacher, Diffprivlib: The IBM differential privacy library. arXiv:1907.02444 [cs.CR] (2019).

[39] K. Gadiya, FIFA 19 complete player dataset (2019); https://www.kaggle.com/datasets/javagarm/fifa-19-complete-player-dataset.

[40] M. Redmond, Communities and Crime Unnormalized dataset, UCI Repository of machine learning databases (2011); https://archive.ics.uci.edu/ml/datasets/communities+and+crime+unnormalized.

[41] T. Dietterich, Musk dataset, version 2, UCI Repository of machine learning databases (1998); https://archive.ics.uci.edu/ml/datasets/Musk+(Version+2).

[42] B. Jayaraman, D. Evans, “Are attribute inference attacks just imputation?” in *Proceedings of the 2022 ACM SIGSAC Conference on Computer and Communications Security* (ACM, 2022), pp. 1569–1582.

[43] S. Mehnaz, S. V. Dibbo, R. De Viti, E. Kabir, B. B. Brandenburg, S. Mangard, N. Li, E. Bertino, M. Backes, E. De Cristofaro, “Are your sensitive attributes private? Novel model inversion attribute inference attacks on classification models” in *31st USENIX Security Symposium (USENIX Security 22)* (USENIX, 2022), pp. 4579–4596.

[44] A. M. G. Salem, A. Bhattacharyya, M. Backes, M. Fritz, Y. Zhang, “Updates-leak: Data set inference and reconstruction attacks in online learning” in *29th USENIX Security Symposium* (USENIX, 2020) pp. 1291–1308.

[45] A. Suri, D. Evans, “Formalizing and estimating distribution inference risks” in *Proceedings on Privacy Enhancing Technologies*, (PoPETS, 2022), vol. 2022, pp. 528–551.

[46] J. Zhou, Y. Chen, C. Shen, Y. Zhang, Property inference attacks against gans. arXiv:2111.07608 (2021).

[47] A. Paszke, S. Gross, F. Massa, A. Lerer, J. Bradbury, G. Chanan, T. Killeen, Z. Lin, N. Gimelshein, L. Antiga, A. Desmaison, A. Köpf, E. Yang, Z. De Vito, M. Raison, A. Tejani, S. Chilamkurthy, B. Steiner, L. Fang, J. Bai, S. Chintala, “Pytorch: An imperative style, high-performance deep learning library” in *Advances in Neural Information Processing Systems 32* (NeurIPS, 2019), pp. 8026–8037.

[48] F. Pedregosa, G. Varoquaux, A. Gramfort, V. Michel, B. Thirion, O. Grisel, M. Blondel, P. Prettenhofer, R. Weiss, V. Dubourg, J. Vanderplas, A. Passos, D. Cournapeau, M. Brucher, M. Perrot, É. Duchesnay, Scikit-learn: Machine learning in python. J. Mach. Learn. Res. 12, 2825–2830 (2011).

[49] X. Shen, S. Diamond, Y. Gu, S. Boyd, “Disciplined convex-concave programming” in *2016 IEEE 55th Conference on Decision and Control (CDC)* (IEEE, 2016), pp. 1009–1014.

