# Supplementary material for: Correlation inference attacks against machine learning models
Source: Sci Adv. 2024 Jul 10;10(28):eadj9260. doi: 10.1126/sciadv.adj9260 (PMC11758436; doi:10.1126/sciadv.adj9260)
Supplement: Supplementary file 1 — Sections S1 to S9 Figs. S1 to S9 Table S1 Algorithm S1 to S3 References [file sciadv.adj9260_sm.pdf]

Science Advances
AAAS

Supplementary Materials for

Correlation inference attacks against machine learning models

Ana-Maria Creţu *et al.*

Corresponding author: Yves-Alexandre de Montjoye, demontjoye@imperial.ac.uk

Sci. Adv. **10**, eadj9260 (2024)
DOI: 10.1126/sciadv.adj9260

This PDF file includes:

Sections S1 to S9
Figs. S1 to S9
Table S1
Algorithm S1 to S3
References

S1 Details of the model-less attack

We present the complete details of the model-less attack, for preciseness and reproducibility. First, we define the N_B classification bins as the following intervals:

$$\begin{aligned}
&\left[\frac{2(b-1)-N_B}{N_B}, \frac{2b-N_B}{N_B}\right), \text{ for } b = 1, \dots, N_B - 1 \\
&\left[\frac{2(b-1)-N_B}{N_B}, \frac{2b-N_B}{N_B}\right], \text{ for } b = N_B
\end{aligned} \tag{4}$$

Denoting by $[m_1, m_2]$ the range of possible values for target correlation $\rho(X_1, X_2)$, our model-less attack predicts the majority bin over this interval, distinguishing three cases:

(C1) The interval is fully included inside a bin, i.e., $(2(b-1)-N_B)/N_B \leq m_1 \leq m_2 < (2b-N_B)/N_B$ for some $b = 1,\ldots,N_B$. We predict the bin b.

(C2) The interval $[m_1, m_2]$ partially covers two bins, but none entirely, i.e., $m_1 < (2b-N_B)/N_B \leq m_2 < (2(b+1)-N_B)/N_B$, for some $b = 1,\ldots,N_B-1$. We predict the bin b if it has higher coverage than $b+1$ (i.e., if $(2b-N_B)/N_B - m_1 > m_2 - (2(b+1)-N_B)/N_B$), and $b+1$ otherwise.

(C3) At least one bin is fully covered by the interval $[m_1, m_2]$. We predict one of the bins that we sample uniformly at random.

S2 Analysis of regions

We analyze in detail the sets of constraints $(\rho(X_1, Y), \rho(X_2, Y))$ satisfying that there is only one possible bin for $\rho(X_1, X_2)$. We consider $0 \leq \theta_1, \theta_2 \leq \pi$ such that $\rho(X_1, Y) = \cos\theta_1$ and $\rho(X_2, Y) = \cos\theta_2$. As a reminder, the $N_B = 3$ classification bins are $[-1, -1/3)$, $[-1/3, 1/3)$, and $[1/3, 1]$ and the interval attainable by $\rho(X_1, X_2)$ is $[\cos(\theta_1+\theta_2), \cos(\theta_1-\theta_2)]$.

- **Negative bin:** $\cos(\theta_1-\theta_2) \leq -\frac{1}{3}$. It follows that $\theta_1 - \theta_2 \geq \arccos(-\frac{1}{3})$ if $\theta_1 \geq \theta_2$ and $\theta_2 - \theta_1 \geq \arccos(-\frac{1}{3})$ otherwise.

- **Low bin:** $-\frac{1}{3} \leq \cos(\theta_1+\theta_2) \leq \cos(\theta_1-\theta_2) < \frac{1}{3}$. We distinguish 4 subcases:

  (I) $\theta_1 \geq \theta_2$ and $0 \leq \theta_1+\theta_2 \leq \pi$. It follows that $\theta_1+\theta_2 \leq \arccos\left(-\frac{1}{3}\right)$ and $\theta_1-\theta_2 > \arccos\left(\frac{1}{3}\right)$.

  (II) $\theta_1 < \theta_2$ and $0 \leq \theta_1+\theta_2 \leq \pi$. It follows that $\theta_1+\theta_2 \leq \arccos\left(-\frac{1}{3}\right)$ and $\theta_2-\theta_1 > \arccos\left(\frac{1}{3}\right)$.

  (III) $\theta_1 \geq \theta_2$ and $\pi \leq \theta_1+\theta_2 \leq 2\pi$. It follows that $\theta_1+\theta_2 \geq \pi + \arccos\left(\frac{1}{3}\right)$ and $\theta_1-\theta_2 > \arccos\left(\frac{1}{3}\right)$.

  (IV) $\theta_1 < \theta_2$ and $\pi \leq \theta_1+\theta_2 \leq 2\pi$. It follows that $\theta_1+\theta_2 \geq \pi + \arccos\left(\frac{1}{3}\right)$ and $\theta_2-\theta_1 > \arccos\left(\frac{1}{3}\right)$.

- **Positive bin:** $\frac{1}{3} < \cos(\theta_1+\theta_2)$ It follows that $\theta_1+\theta_2 < \arccos(\frac{1}{3})$ if $\theta_1+\theta_2 \leq \pi$ and to $\theta_1+\theta_2 > \pi + \arccos(-\frac{1}{3})$ if $\pi \leq \theta_1+\theta_2 \leq 2\pi$.

S3 Algorithm to sample a valid correlation matrix

Alg. S1 details an implementation of the algorithm to sample a valid correlation matrix by Numpacharoen and Atsawarungruangkit [28]. We refer the reader to the original work for a slightly modified version which correctly handles the numerically unstable cases when the range of attainable values for $c_{i,j}$ is very small.

Algorithm S1 SAMPLECORRMATRIX [28]. We highlight in red the statement which we modify when calling this algorithm inside Alg. 1 of the main paper and Alg. S2. The modified statement for each case is provided in Sec. 4.1.1 and is omitted here for brevity. Code comments are written in blue.

Inputs:
  n: Number of variables.
Output:
  $C \in \mathbb{R}^{n \times n}$: A valid correlation matrix.
Initialize:
  $C \leftarrow 0$; $B \leftarrow 0$
`// Randomly initialize the first column.`
for $i \in \{2, \ldots, n\}$ **do**
  $c_{i,1} \leftarrow \mathcal{U}(-1, 1)$ `//` $\cos \theta_{i,1}$
  $b_{i,1} \leftarrow c_{i,1}$
  $b_{i,j} \leftarrow \sqrt{1 - c_{i,1}^2}$ for $j \in \{2, \ldots, i\}$ `//` $\sin \theta_{i,1}$
end for
for $i \in \{2, \cdots, n\}$ **do**
  for $j \in \{1, \cdots, i\}$ **do**
    `// Compute the bounds` $m_{i,j} \pm l_{i,j}$.
    $m_{i,j} \leftarrow B_{i,1:j-1}(B_{j,1:j-1})^T$
    $l_{i,j} \leftarrow b_{i,j} b_{j,j}$
    `// Sample uniformly within bounds.`
    $c_{i,j} \leftarrow \mathcal{U}(m_{i,j} - l_{i,j}, m_{i,j} + l_{i,j})$
    `// Update` B.
    $aux \leftarrow \frac{c_{i,j} - m_{i,j}}{l_{i,j}}$ `//` $\cos\ \theta_{i,j}$.
    $b_{i,j} \leftarrow b_{i,j} * aux$
    $b_{i,k} \leftarrow \sqrt{1 - aux^2}$ for $k \in \{j+1, \ldots, n\}$
  end for
end for
$C \leftarrow C + C^T + I_n$

Alternative approaches. The spherical parametrization of correlation matrices provides a principled and effective approach for sampling valid correlation matrices. In contrast, the characterization given by properties **P1-P4** does not easily translate into an effective algorithm for constructing correlation matrices. In preliminary experiments, we framed the correlation matrix generation problem as a constrained optimization program. We initialized a $n \times n$ matrix by setting each element uniformly at random between -1 and 1, then projected it into the space of positive semi-definite matrices having all diagonal entries equal to 1 and all elements between -1 and 1. We used the DCCP library [37]. Due to numerical instability, the resulting matrices often did not satisfy property **P4**, as they had at least one negative eigenvalue (40% of the time for $n = 4$ variables and decreasing steadily with n). As a result, several instances of the optimization program are required to generate one valid correlation matrix, and their number increases with the number of variables n. In constrast, Alg. S2 succeeds 100% of the time, regardless of the number of variables. Furthermore, our approach is 10 times faster than one instance of the optimization program. We also found that the correlations generated by the optimization program are less likely to cover the entire range of values that are theoretically attainable.

S4 Algorithm to sample a correlation matrix under scenario S1

Alg. S2 is the implementation of the algorithm to sample a correlation matrix using the default scenario S1. It uses Alg. S1 while carefully applying the random permutation according to the constraints from the S1 scenario.

S5 Algorithm to sample from Gaussian copulas

Alg. S3 details a procedure to obtain a sample from a n-dimensional Gaussian copulas distribution parametrized by a covariance matrix Σ and one-way marginals $F_1, \ldots, F_n$.

S6 Experimental setup

S6.1 Target models

The target models we study in this paper are the Logistic Regression (LR) and the Multilayer Perceptron (MLP).

Algorithm S2 SampleCorrMatrix-AttackS1. Code comments are written in blue.

Inputs:
n: Number of variables.
$constraints = (\rho(X_1, Y), \rho(X_2, Y))$: Correlation constraints imposed by the adversary's knowledge.
Output:
$C \in \mathbb{R}^{n \times n}$: A valid correlation matrix satisfying $c_{1,n} = \rho(X_1, Y)$ and $c_{2,n} = \rho(X_2, Y)$.
$C \leftarrow \textsc{SampleCorrMatrix}(n, constraints)$
`// Reorder variables as` $X_1, X_2, X_{\sigma(3)}, \ldots, X_{\sigma(n-1)}, Y$.
$\sigma \leftarrow [2, 3] + random\_permutation([4, \ldots, n]) + [1]$
$C \leftarrow reorder\_columns(reorder\_rows(C, \sigma), \sigma)$

Algorithm S3 SampleFromGaussianCopulas. Code comments are written in blue.

Inputs:
n: Number of variables.
$\Sigma \in \mathbb{S}_+^d$: A valid covariance matrix.
$F_1, \ldots, F_n$: One-way marginals.
Output:
A sample $z = (z_1, \ldots, z_n)$ from a Gaussian copula distribution parametrized by $F_1, \ldots, F_n$ and Σ.
$A \leftarrow$ **CholeskyDecomposition**(C) `//` $C = A^t \cdot A$
$Z \sim \mathcal{N}(0, I_n)$
$X \leftarrow A^T Z$
for $i = 1, \ldots, n$ **do**
  $z_i \leftarrow F_i^{-1}(X_i)$
end for
$z \leftarrow (z_1, \ldots, z_n)$

For the LR models, we use the implementation provided by the Scikit-learn 0.24.1 [30] library with default parameters. For the MLP models, we implement in Pytorch 1.10.0 [29] an architecture consisting of two hidden layers of sizes 20 and 10 with ReLU nonlinearity. In the experiments on synthetic data (Sec. 2.3.1- 2.3.3), we train the models using gradient descent. In the experiments on real-world datasets (Sec. 2.3.4), we instead train the models using mini-batch gradient descent with a batch size of 128, as this

leads to better target model accuracy. The models are trained on 90% of the samples for up to 100 epochs, stopping the models after 5 epochs of non-improving accuracy on the remaining 10%. We use a learning rate of $\eta = 0.05$.

S6.2 Datasets

We use three datasets in our real-world evaluation (Sec. 2.3.4).

Fifa19 [17] contains 18207 records of 89 attributes each, describing physical attributes and performance statistics of football players. We discard the categorical attributes, keeping the continuous and discrete ordinal variables. We select the *Value* attribute, representing the amount of money a club payed for a player, as the output variable, discarding the other attributes predictive of it that would make classification trivial (e.g., $Wage$). We then remove the records with missing values, leaving us with 15917 records. We binarize the output variable by mapping it to $Y = 1$ if larger than the median value (> 0.74M) and to $Y = 0$ otherwise. Finally, we remove the duplicate attributes, which leaves us with 53 input attributes.

Communities and Crime [33] contains 2215 records of 147 attributes each, describing socio-economic, law enforcement, and crime statistics on communities in the US. We select the number of murders as the output variable of our machine learning models, discarding the other columns predictive of it (i.e., relating explicitly to crime). As input attributes, we select the 101 continuous and discrete ordinal attributes from the dataset that do not have missing values, discarding the rest. We binarize the number of murders by mapping it to $Y = 1$ if is at least 1, and to $Y = 0$ otherwise.

Musk (v2) [13] contains 6598 records of 166 attributes each, describing different conformations of 165 molecules, together with a human expert-assigned label of "musk" or "non-musk". We train the models to classify conformations as "musk" ($Y = 1$) or "non-musk" ($Y = 0$). The dataset being heavily imbalanced with respect to the two classes, we balance it for simplicity, leaving us with 2034 records.

S6.3 Attack parameters

Unless otherwise specified, throughout the paper we will consider the default attacker **S2**. Given a target model $\mathcal{M}_T$, we train a meta-classifier on outputs extracted from $k = |D_{\text{meta}}|$ models. We generate $K = 5000$ synthetic datasets for target models $\mathcal{M}_T$ trained on $n = 3, 4$ and 5 variables and $K = 10000$ synthetic datasets for $n = 6$ to 10 variables. In the experiments

on synthetic data, the models are all trained on 1000 samples and the target model test accuracy is computed on 500 unseen samples. In the experiments on real-world datasets, the target models are trained on all the samples available ($|D_T| = 2215$ for Communities and crime and $|D_T| = 2034$ for Musk), except for Fifa19, where we sample $|D_T| = 2000$ records uniformly without replacement to keep the dataset sizes similar. The synthetic datasets are always generated to have a number of samples equal to the target dataset size $|D_T|$. We run the ModifyCorrelationConstraints heuristic (Alg. 3) using parameter values $S = 100$ and $N_D = |D_T|$, for a maximum of $M = 10$ iterations and with an error tolerance of $e = 0.01$. We set the size of the query dataset D_{query} equal to $Q = 100$, and discuss in Sec 2.3.3 the impact of this parameter.

We use a Logistic Regression as the meta-classifier for LR models, as it leads to similar accuracy as the MLP but takes less time to train. We use an MLP as the meta-clasifier for MLP models, trained the same as stated in Sec. S6.1 except that we use a batch size of 128, a learning rate of $\eta = 0.001$, $\mathbb{L}_2$ weight decay of 0.01, and early stopping after 10 epochs of non-improving accuracy on 10% of held-out samples.

S6.4 Model-less attack performance: number of samples

Throughout the experiments, for a fair comparison between the model-less and model-based attacks, we have used the same number of samples for the model-less and model-based attack, K. We have opted for relatively large values of K, in the order of thousands (see Sec. S6.3 for the values used). This is because the model-based attack, being machine learning-based, needs more samples to converge. We here examine how the performance of the model-less attack varies with the number of samples K. We compute the accuracy of the model-less attack over $T = 1000$ correlation matrices of $n = 3$ variables using $K \in \{1, 2, 5, \ldots, 100, 200, 500, 1000\}$.

Fig. S1 shows that the accuracy of the model-less attack converges very quickly as a function of the number of samples. This is because the model-less attack predicts which of the $N_B = 3$ bins is most covered by the segment $[\min(samples), \max(samples)]$, as described in Sec. 2.2 - Model-less attack and Appendix S1 and this prediction is very robust to the number of samples used.

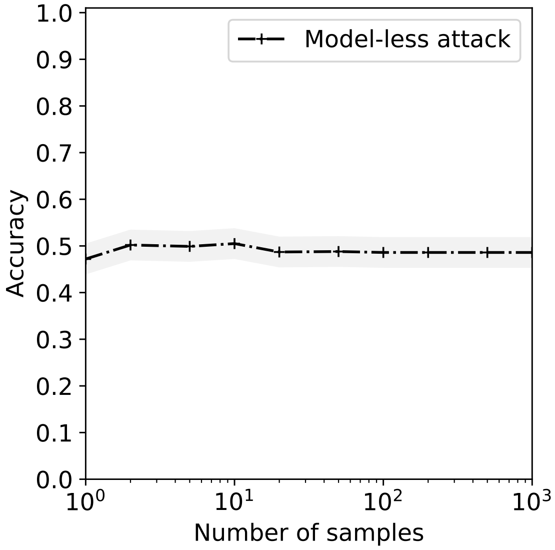

Figure S1: **Impact of the number of samples K on the model-less attack performance.** We report the accuracy (with 95% confidence interval) over 1000 target models.

S7 White-box results

We evaluate the performance of white-box correlation inference attacks using the model weights as features. The weights of the Logistic Regression are its coefficients (including the bias). For the MLP, we explore two options: (1) using the *raw weights* (coefficients of the linear layers, including the bias), flattened and concatenated into a single vector and (2) the *canonical weights*, extracted in the same way from the model after sorting the neurons in each layer according to the sum of weights [18].

Fig. S2 shows that the weights of Logistic Regression models yield similar performance to the confidence scores (95.1% vs 95.6%). This is likely due to the small number of weights (three in total), whose information we believe to be redundant with the one contained in the confidence scores extracted from $Q = 100$ records.

Fig. S3 shows that the raw weights of MLP models do not leak more information than the model-less attack (55.0%). This negative result is in line with previous findings from the property inference literature [18, 47] and can be attributed to the permutation equivalence property of MLPs [18], whereby neurons of internal layers can be permuted without changing the

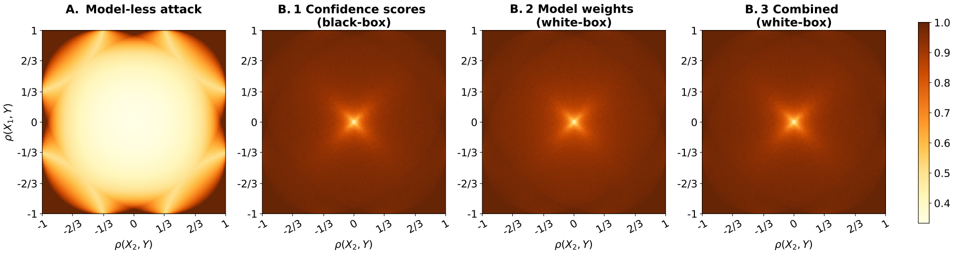

Figure S2: **Logistic regression: Comparison between black-box and white-box correlation inference attacks.** We report results for our model-less attack (A), our black-box model-based attack using as features the confidence scores (B. 1), and our white-box model-based attacks using as features the model weights (B. 2) or the combined weights and confidence scores (B. 3).

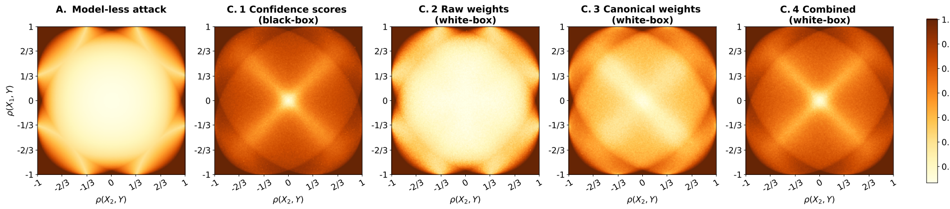

Figure S3: **Multilayer perceptron (MLP): Comparison between black-box and white-box correlation inference attacks.** We report results for our model-less attack (A), our black-box model-based attack using as features the confidence scores (C. 1), and our white-box model-less attacks using as features the raw model weights (C. 2), the canonical weights (i.e., the raw weights mapped to a canonical representation [18], C. 3), or the combined canonical weights and confidence scores (C. 4).

underlying function. As a result, a meta-classifier operating on inputs in their default ordering (with $k!$ equivalent orderings being possible in each layer, where k is the number of neurons in the layer) needs to learn a more difficult task. We show that the canonical weights yield much better performance (65.5%), albeit lower than the confidence scores.

On both models, combining the model weights and the confidence scores (Fig.S2-B.3 and Fig. S3-C.4) does not yield better results than using the confidence scores alone.

We refer the reader to Sec. 3 for results of our white-box correlation inference attacks on MLP models when the attacker is assumed to know the seed used to initialize the target model's training algorithm.

S8 Additional results on synthetic datasets

Fig. S7 shows the impact of the largest constraint $\max(|\rho(X_1, Y), \rho(X_2, Y)|)$ on the accuracy of our model-based and model-less attack. For completeness, we also include results on the impact of the average constraint $(|\rho(X_1, Y), \rho(X_2, Y)|)/2$ (Fig. S8) and of the smallest constraint $\min(|\rho(X_1, Y), \rho(X_2, Y)|)$ (Fig. S9).

Fig. S4 shows results of our correlation inference attack using $N_B = 5$ bins.

Fig. S5 shows the impact of mitigation techniques against our attack targeting MLP models.

Fig. S6 shows the impact of the number of sub-intervals G over which the one-way marginals released to the attacker are computed on the accuracy of our attack.

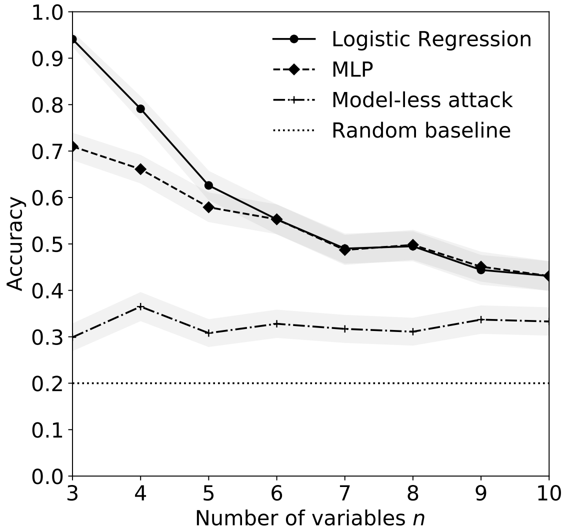

Figure S4: **Attack accuracy under scenario S2 and $N_B = 5$ correlation bins for different number of variables in the dataset n.** We report the accuracy (with 95% confidence interval) over 1000 target models.

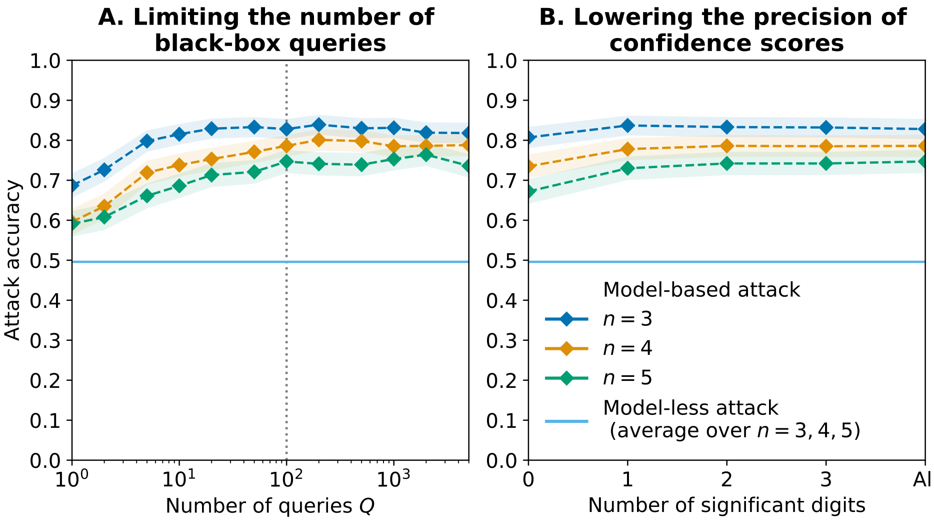

Figure S5: **Impact of mitigations on the accuracy of our attack against MLP models.** We report results for two mitigations: limiting the number of black-box queries (A) and lowering the precision of confidence scores (B). We report the accuracy (with 95% confidence interval) of our model-less and model-based attacks over 1000 targets models for $n \in \{3, 4, 5\}$ variables.

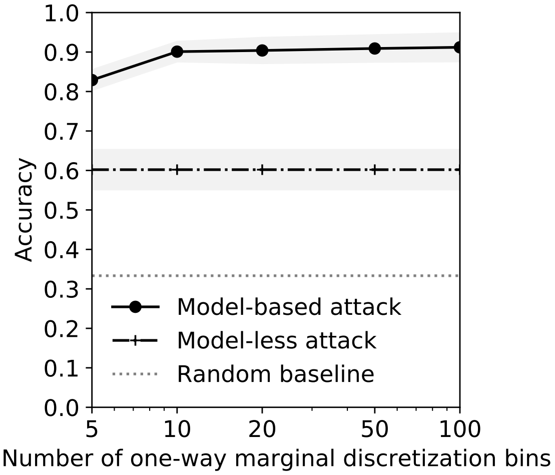

Figure S6: **Fifa19: Impact of the granularity of one-way marginals available to the attacker.** We report the attack accuracy (mean and standard deviation) on logistic regression models as we vary the number of discretization bins applied to the one-way marginals.

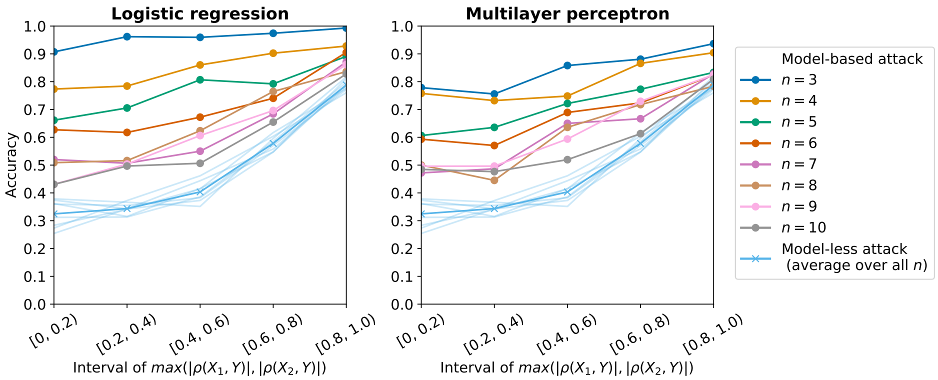

Figure S7: **Impact of the largest constraint** $\max(|\rho(X_1, Y)|, |\rho(X_2, Y)|)$ **on the attack accuracy for different number of variables** n**.** We compare the results of our model-based and model-less attacks on logistic regression (left) and MLP models (right).

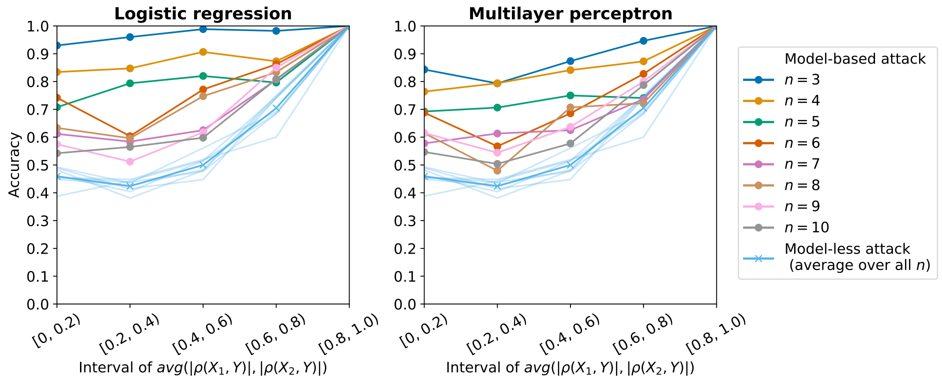

Figure S8: **Impact of the average constraint** $(|\rho(X_1, Y)| + |\rho(X_2, Y)|)/2$ **on the attack accuracy for different number of variables** n**.** We compare the results of our model-based and model-less attacks on logistic regression (left) and MLP models (right).

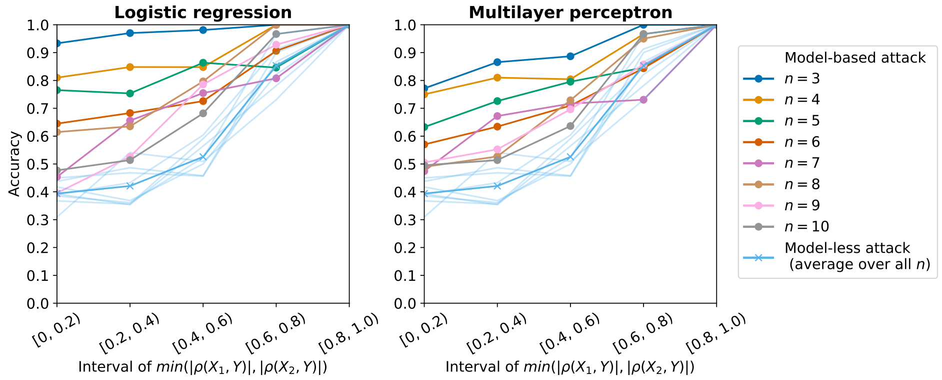

Figure S9: **Impact of the smallest constraint** $\min(|\rho(X_1, Y)|, |\rho(X_2, Y)|)$ **on the attack accuracy for different number of variables** n**.** We compare the results of our model-based and model-less attacks on logistic regression (left) and MLP models (right).

S9 Attribute inference attack: details and baselines

We implement CI-AIA using the same parameters as in the Supplementary Materials S6.3, $S' = 1000$ synthetic datasets, $G = 100$ bins, and $m_i = 2$ and $\delta_i = 0.5$ for every $i = 1, \ldots, n-1$.

We now describe the five attacks from the previous works against which we compare our CI-AIA method. To perform attribute inference over a continuous sensitive attribute, we divide the attribute range into G bins of equal length, infer the most likely bin using a given attack, then return a uniform sample from the bin as the final prediction. Given a candidate record $(x_1^g, x_2, \ldots, x_{n-1}, y)$ where the value of the unknown sensitive attribute x_1 is substituted with a sample x_1^g of the g-th candidate bin, we will denote by V_y^g the confidence that the candidate record is classified with the target label y, i.e., the l-th element of output vector $\mathcal{M}_T(x_1^g, x_2, \ldots, x_{n-1})$.

1. **Fredrikson et al. [16]** The attack infers the most likely bin $g = 1, \ldots, G$ as per the probability $\Pr(x_1^g)\Pr(\hat{y} = y | \arg\max_{l=1}^{L} \mathcal{M}_T(x_1^g, x_2, \ldots, x_{n-1}) = \hat{y})$, where x_1^g is a value sampled uniformly at random in the g-th subinterval. The second term is approximated using the confusion matrix of the target model, assumed by Fredrikson et al. [16] to be known by the attacker.

2. **CSMIA (Mehnaz et al. [26])** The attack sets the sensitive attribute value of the partial record to each possible value (here, one of G bins) and retrieves the model prediction $\hat{y}^g = \mathcal{M}_T(x_1^g, x_2, \ldots, x_{n-1}), g = 1, \ldots, G$. Note that we represent a bin using a uniform sample in the bin x_1^g. Then, the attack distinguishes three cases: (1) if $\hat{y}^g = y$ for only one value of g, the approach returns x_1^g, (2) if $\hat{y}^g = y$ for more than one value of g, the approach returns x_1^g on which the model is the most confident, and (3) if $\hat{y}^g \neq y$ for all values of g, the approach returns x_1^g on which the model is the least confident.

3. **Yeom et al. [46]** The attack returns the sensitive attribute value with the largest prior $\Pr(x_1^g)$ that passes a membership inference test. More specifically, the attack uses a membership oracle $\mathbb{O}$ that returns 1 if $(x_1^g, x_2, \ldots, x_{n-1}, y) \in D_T$ and 0 otherwise. The attack returns $\mathrm{argmax}_g \Pr(x_1^g) \cdot \mathbb{O}(\mathcal{M}_T, (x_1^g, x_2, \ldots, x_{n-1}))$ (where x_1^g is sampled uniformly at random in the g-th bin). We use the threshold-based membership oracle of Yeom et al. [46], that returns 1 if and only if the

model's confidence on the target record is larger than a threshold τ. We use $\tau = 0.5$.

4. **Jayaraman and Evans [24]** removes the need for an oracle in the attack by Yeom et al. [46], proposing two variants. The first one is CAI, which returns $\arg\max_g V_y^g$. The second one is WCAI, which returns $\arg\max_g \Pr(x_1^g | x_2, \ldots, x_{n-1}) V_y^g$, making use of the conditional probability of the sensitive attribute given the non-sensitive attributes. In their work, this quantity is estimated empirically by fitting a machine learning model to infer X_1 based on $X_2, \ldots, X_{n-1}$. The model is fitted on auxiliary data drawn from a similar distribution as the private dataset. As we here study a weaker adversary lacking access to auxiliary data, under our assumptions the attack would simply become $\arg\max_g \Pr(x_1^g) V_y^g$.

Our correlation inference-based attribute inference attack (CI-AIA) is fundamentally different from Jayaraman and Evans's AIA [24]. This is because we do not assume the adversary to know the data distribution via access to auxiliary data. Instead, we extract information about the distribution from the model (the correlation between the input variables) and use it to generate synthetic data. In spite of this fundamental difference, the WCAI formula for inferring the sensitive attribute given the partial record is similar to step 3 of our CI-AIA attack. Recall that in our step 3, we retrieve the average sensitive attribute value x_1^g among all synthetic records which approximately match the partial record $(x_2, x_3, ..., x_{n-1})$ and the label y. Our step 3 can thus be seen as equivalent to estimating $E(x_1 | x_2, ..., x_{n-1}, y)$, while WCAI returns $\arg\max_g p(x_1^g | x_2, ..., x_{n-1}) V_y^g$.

We study if our CI-AIA can be further improved by applying the WCAI formula instead of our step 3 (described in Sec. 4.2) to the synthetic data generated conditionally on the correlations inferred using our attack. For completeness, we explore different variants the WCAI formula.

1. Variant 1: We trained a regression model for inferring x_1 given $x_2, \ldots, x_{n-1}$, like [24]. The model is trained on the same synthetic data – generated conditionally on the correlations inferred from the model – as our attack, instead of auxiliary data [24] which our attacker does not have.

2. Variant 2: This is the same as variant 1, except that to understand the impact of conditioning on y – which our step 3 does while [24] does not – we trained a regression model for inferring x_1 given $x_2, x_3, \ldots, x_{n-1}$, **and** y.

3. Variant 3: This is the same as variant 1, except that we multiply $p(x_1^g|x_2,\ldots,x_{n-1})$ by the model's confidence on label y, V_y^g, to understand if V_y^g helps. This is the closest to the formula of [24], the only difference being how $p(x_1^g|x_2,\cdots,x_{n-1})$ is estimated. Jayaraman and Evans [24] uses a neural network classifier which outputs probabilities, while we use a regression since our attribute x_1 is continuous. As the regression model does not output a probability, we model it as the probability of error using a standard normal distribution $p(x_1^g|x_2,\ldots,x_{n-1}) \sim e^{-\frac{(x_1^*-x_1^g)^2}{2}}$, where x_1^* denotes the model's prediction on the partial record $(x_2,\ldots,x_{n-1})$.

4. Variant 4: This is the same as variant 3, except that to understand the impact of conditioning on y – which we do while [24] does not – we trained a regression model for inferring x_1 given $x_2, x_3, \ldots, x_{n-1}$ **and** y.

Table S1 shows that none of the variants of our CI-AIA attack achieve better performance than our original attack. In particular, our CI-AIA achieves the same accuracy (49.7%) as the WCAI formula [24] applied to the synthetic data generated using steps 1 and 2 of our attack (49.8% - Variant 3). This means that once correlations have been extracted and synthetic data has been generated conditionally on these correlations using steps 1 and 2 of our attack, either our step 3 or Jayaraman and Evans's [24] formula can be used to achieve similar results. Another interesting finding is that using y in the prediction leads to better performance than not using y at all. The AIA accuracy is 49.5% when x_1 is inferred conditionally on y in addition to the other variables (Variant 2), 49.8% when the prediction uses the model's confidence on label y (Variant 3) and 46.7% when y is not used at all (Variant 1). Using y by both conditioning on it and via the model's confidence (Variant 4) does not significantly improve AIA performance.

**Method**	**Accuracy**
(Ours) CI-AIA	49.7 ± 1.0
Variant 1	46.7 ± 1.0
Variant 2	49.5 ± 1.0
Variant 3 (closest formula to [24])	49.8 ± 1.0
Variant 4	50.5 ± 0.9

Table S1: **Comparison between our CI-AIA and attacks obtained by substituting Jayaraman and Evans's [24] prediction rule to step 3 of CI-AIA.** We report the attack accuracy averaged over 1000 runs (with 95% confidence interval) on the Fifa19 dataset.

REFERENCES AND NOTES

1. S. Qummar, F. G. Khan, S. Shah, A. Khan, S. Shamshirband, Zia U. Rehman, I. A. Khan, W. Jadoon. A deep learning ensemble approach for diabetic retinopathy detection *IEEE Access* **7**, 150530–150539 (2019).

2. Y. Wu, M. Schuster, Z. Chen, Q. V. Le, M. Norouzi, W. Macherey, M. Krikun, Y. Cao, Q. Gao, K. Macherey, J. Klingner, A. Shah, M. Johnson, X. Liu, Ł. Kaiser, S. Gouws, Y. Kato, T. Kudo, H. Kazawa, K. Stevens, G. Kurian, N. Patil, W. Wang, C. Young, J. Smith, J. Riesa, A. Rudnick, O. Vinyals, G. Corrado, M. Hughes, J. Dean, Google's neural machine translation system: Bridging the gap between human and machine translation. arXiv:1609.08144 (2016).

3. Siri Team (Apple), Deep learning for siri's voice: On-device deep mixture density networks for hybrid unit selection synthesis (2017); https://machinelearning.apple.com/research/siri-voices.

4. Amazon Rekognition, Moderating content; https://docs.aws.amazon.com/rekognition/latest/dg/moderation.html.

5. H. Chen, O. Engkvist, Y. Wang, M. Olivecrona, T. Blaschke, The rise of deep learning in drug discovery. *Drug Discov. Today* **23**, 1241–1250, 2018.

6. A. Gordo, J. Almazan, J. Revaud, D. Larlus, "Deep image retrieval: Learning global representations for image search" in *Computer Vision–ECCV 2016: 14th European Conference, Amsterdam, The Netherlands, October 11–14, 2016, Proceedings, Part VI 14* (Springer, 2016), pp. 241–257.

7. I. Goodfellow, Y. Bengio, A. Courville, *Deep Learning* (MIT Press, 2016).

8. A. Alvi, P. Kharya, Using deepspeed and megatron to train megatron-turing nlg 530b, the world's largest and most powerful generative language model (2021); www.microsoft.com/en-us/research/blog/using-deepspeed-and-megatron-to-train-megatron-turing-nlg-530b-the-worlds-largest-and-most-powerful-generative-language-model/.

9. M. Veale, R. Binns, L. Edwards, Algorithms that remember: Model inversion attacks and data protection law. *Philos. Trans. R. Soc. A Math. Phys. Eng. Sci.* **376**, 20180083 (2018).

10. G. Ateniese, L. V. Mancini, A. Spognardi, A. Villani, D. Vitali, G. Felici, Hacking smart machines with smarter ones: How to extract meaningful data from machine learning classifiers. *Int. J. Secur. Netw.* **10**, 137–150 (2015).

11. B. Balle, G. Cherubin, J. Hayes, "Reconstructing training data with informed adversaries" in *2022 IEEE Symposium on Security and Privacy (SP)* (IEEE, 2022), pp. 1138–1156.

12. N. Carlini, F. Tramèr, E. Wallace, M. Jagielski, A. Herbert-Voss, K. Lee, A. Roberts, T. Brown, D. Song, Ú. Erlingsson, A. Oprea, C. Raffel, "Extracting training data from large language models" in *USENIX Security Symposium*, vol. 6 (USENIX, 2021).

13. M. Fredrikson, S. Jha, T. Ristenpart, "Model inversion attacks that exploit confidence information and basic countermeasures" in *Proceedings of the 22nd ACM SIGSAC Conference on Computer and Communications Security* (ACM, 2015), pp. 1322–1333.

14. M. Fredrikson, E. Lantz, S. Jha, S. Lin, D. Page, T. Ristenpart, "Privacy in pharmacogenetics: An end-to-end case study of personalized warfarin dosing" in *23rd USENIX Security Symposium (USENIX Security 14)* (USENIX, 2014), pp. 17–32.

15. R. Shokri, M. Stronati, C. Song, V. Shmatikov, "Membership inference attacks against machine learning models" in *2017 IEEE Symposium on Security and Privacy (SP)* (IEEE, 2017), pp. 3–18.

16. N. Homer, S. Szelinger, M. Redman, D. Duggan, W. Tembe, J. Muehling, J. V. Pearson, D. A. Stephan, S. F. Nelson, D. W. Craig. Resolving individuals contributing trace amounts of DNA to highly complex mixtures using high-density SNP genotyping microarrays. *PLOS Genet.* **4**, e1000167 (2008).

17. N. Carlini, S. Chien, M. Nasr, S. Song, A. Terzis, F. Tramer, "Membership inference attacks from first principles" in *2022 IEEE Symposium on Security and Privacy (SP)* (IEEE, 2022), pp. 1897–1914.

18. C. A. Choquette-Choo, F. Tramer, N. Carlini, N. Papernot, "Label-only membership inference attacks" in *International Conference on Machine Learning* (PMLR, 2021), pp. 1964–1974.

19. B. Jayaraman, D. Evans, "Evaluating differentially private machine learning in practice" in *28th USENIX Security Symposium (USENIX Security 19)*, (USENIX, 2019), pp. 1895–1912.

20. K. Leino, M. Fredrikson, "Stolen memories: Leveraging model memorization for calibrated white-box membership inference" in *29th USENIX security symposium (USENIX Security 20)* (USENIX, 2020), pp. 1605–1622.

21. M. Nasr, R. Shokri, A. Houmansadr, "Comprehensive privacy analysis of deep learning: Passive and active white-box inference attacks against centralized and federated learning" in *2019 IEEE Symposium on Security and Privacy (SP)* (IEEE, 2019), pp. 739–753.

22. A. Salem, Y. Zhang, M. Humbert, P. Berrang, M. Fritz, M. Backes, Ml-leaks: Model and data independent membership inference attacks and defenses on machine learning models. arXiv:1806.01246 (2018).

23. S. Truex, L. Liu, M. E. Gursoy, Lei Yu, W. Wei, Demystifying membership inference attacks in machine learning as a service. *IEEE Trans. Serv. Comput.* **14**, 2073–2089 (2021).

24. S. Yeom, I. Giacomelli, M. Fredrikson, S. Jha, "Privacy risk in machine learning: Analyzing the connection to overfitting" in *2018 IEEE 31st Computer Security Foundations Symposium (CSF)* (IEEE, 2018), pp. 268–282.

25. J. Buolamwini, T. Gebru, "Gender shades: Intersectional accuracy disparities in commercial gender classification" in *Conference on Fairness, Accountability and Transparency* (PMLR, 2018), pp. 77–91.

26. K. Ganju, Q. Wang, W. Yang, C. A. Gunter, N. Borisov, "Property inference attacks on fully connected neural networks using permutation invariant representations" in *Proceedings of the 2018 ACM SIGSAC Conference on Computer and Communications Security* (ACM, 2018), pp. 619–633.

27. W. Zhang, S. Tople, O. Ohrimenko, “Leakage of dataset properties in multi-party machine learning” in *USENIX Security Symposium* (USENIX, 2021), pp. 2687–2704.

28. Patrik Waldmann. On the use of the pearson correlation coefficient for model evaluation in genome-wide prediction. *Front. Genet.* **10**, 899 (2019).

29. H. Zhou, Z. Deng, Y. Xia, M. Fu, A new sampling method in particle filter based on pearson correlation coefficient. *Neurocomputing* **216**, 208–215 (2016).

30. European Commission, “On artificial intelligencea european approach to excellence and trust” (European Commission, 2020); https://ec.europa.eu/info/sites/default/files/commission-white-paper-artificial-intelligence-feb2020_en.pdf.

31. J. C. Pinheiro, D. M. Bates, Unconstrained parametrizations for variance-covariance matrices. *Stat. Comput.* **6**, 289–296 (1996).

32. K. Numpacharoen, A. Atsawarungruangkit, Generating correlation matrices based on the boundaries of their coefficients. *PLOS ONE* **7**, e48902 (2012).

33. M. Sklar, “Fonctions de répartition à n dimensions et leurs marges”, in *Annales de l’ISUP*, vol. 8 (HAL Open Science, 1959), pp. 229–231.

34. Q. Xia, Calculating correlation coefficient for gaussian copula. arXiv:1608.00738 (2016).

35. A. Choromanska, M. Henaff, M. Mathieu, G. B. Arous, Y. LeCun, “The loss surfaces of multilayer networks” in *Artificial Intelligence and Statistics* (PMLR, 2015), pp. 192–204.

36. C. Dwork, F. McSherry, K. Nissim, A. Smith, “Calibrating noise to sensitivity in private data analysis” in *Theory of Cryptography: Third Theory of Cryptography Conference, TCC 2006, New York, NY, USA, March 4–7, 2006. Proceedings 3* (Springer, 2006), pp. 265–284.

37. K. Chaudhuri, C. Monteleoni, A. D. Sarwate, Differentially private empirical risk minimization. *J. Mach. Learn. Res.* **12**, 1069–1109 (2011).

38. N. Holohan, S. Braghin, P. M. Aonghusa, K. Levacher, Diffprivlib: The IBM differential privacy library. arXiv:1907.02444 [cs.CR] (2019).

39. K. Gadiya, FIFA 19 complete player dataset (2019); www.kaggle.com/datasets/karangadiya/fifa19.

40. M. Redmond, Communities and Crime Unnormalized dataset, UCI Repository of machine learning databases (2011); https://archive.ics.uci.edu/ml/datasets/communities+and+crime+unnormalized.

41. T. Dietterich, Musk dataset, version 2, UCI Repository of machine learning databases (1998); https://archive.ics.uci.edu/ml/datasets/Musk+(Version+2).

42. B. Jayaraman, D. Evans, "Are attribute inference attacks just imputation?" in *Proceedings of the 2022 ACM SIGSAC Conference on Computer and Communications Security* (ACM, 2022), pp. 1569–1582.

43. S. Mehnaz, S. V. Dibbo, R. De Viti, E. Kabir, B. B. Brandenburg, S. Mangard, N. Li, E. Bertino, M. Backes, E. De Cristofaro, "Are your sensitive attributes private? Novel model inversion attribute inference attacks on classification models" in *31st USENIX Security Symposium (USENIX Security 22)* (USENIX, 2022), pp. 4579–4596.

44. A. M. G. Salem, A. Bhattacharyya, M. Backes, M. Fritz, Y. Zhang, "Updates-leak: Data set inference and reconstruction attacks in online learning" in *29th USENIX Security Symposium* (USENIX, 2020), pp. 1291–1308.

45. A. Suri, D. Evans, "Formalizing and estimating distribution inference risks" in *Proceedings on Privacy Enhancing Technologies*, vol. 2022 (2022), pp. 528–551.

46. J. Zhou, Y. Chen, C. Shen, Y. Zhang, Property inference attacks against gans. arXiv:2111.07608 (2021).

47. A. Paszke, S. Gross, F. Massa, A. Lerer, J. Bradbury, G. Chanan, T. Killeen, Z. Lin, N. Gimelshein, L. Antiga, A. Desmaison, A. Köpf, E. Yang, Z. De Vito, M. Raison, A. Tejani, S.

Chilamkurthy, B. Steiner, L. Fang, J. Bai, S. Chintala, "Pytorch: An imperative style, high-performance deep learning library" in *Advances in Neural Information Processing Systems 32* (NeurIPS, 2019), pp. 8026–8037.

48. F. Pedregosa, G. Varoquaux, A. Gramfort, V. Michel, B. Thirion, O. Grisel, M. Blondel, P. Prettenhofer, R. Weiss, V. Dubourg, J. Vanderplas, A. Passos, D. Cournapeau, M. Brucher, M. Perrot, É. Duchesnay, Scikit-learn: Machine learning in python. *J. Mach. Learn. Res.* **12**, 2825–2830 (2011).

49. X. Shen, S. Diamond, Y. Gu, S. Boyd, "Disciplined convex-concave programming" in *2016 IEEE 55th Conference on Decision and Control (CDC)* (IEEE, 2016), pp. 1009–1014.
